# Intact Proteoform Analysis by Capillary Electrophoresis–Mass Spectrometry. Are We There Yet?

**DOI:** 10.1002/anie.202518366

**Published:** 2025-12-18

**Authors:** Noah Gould, Qianjie Wang, Jeffrey N. Agar, Jennifer S. Brodbelt, Daoyang Chen, Kellye A. Cupp‐Sutton, Elena Domínguez‐Vega, Fei Fang, Marianne Fillet, Matthew S Fischer, Attila Gaspar, Ying Ge, Marie‐Jia Gou, Michal Greguš, Christoph Gstöttner, Narmin Hamidli, Amanda Helms, Md Amin Hossain, Kyle J. Juetten, Neil L. Kelleher, Tobias Kraus, Eli J. Larson, J. Scott Mellors, Cynthia Nagy, Christian Neusüß, Erin A. Redman, Jasmin Schairer, Si Wu, Tian Xu, Zhitao Zhao, Guijie Zhu, Alexander R. Ivanov, Kevin Jooß, Liangliang Sun

**Affiliations:** ^1^ Barnett Institute of Chemical and Biological Analysis and Department of Chemistry & Chemical Biology Northeastern University Boston MA USA; ^2^ Department of Chemistry Michigan State University East Lansing MI USA; ^3^ Department of Chemistry University of Texas Austin TX USA; ^4^ Discovery Biologics Merck Research Laboratory USA; ^5^ Department of Chemistry and Biochemistry University of Alabama Tuscaloosa AL USA; ^6^ Center for Proteomics and Metabolomics Leiden University Medical Center Leiden The Netherlands; ^7^ Laboratory for the Analysis of Medicines Center for Interdisciplinary Research on Medicines (CIRM) University of Liege Quartier Hospital Liege Belgium; ^8^ Department of Chemistry University of Wisconsin‐Madison Madison Wisconsin 53706 USA; ^9^ Department of Cell and Regenerative Biology and Human Proteomics Program University of Wisconsin‐Madison Madison Wisconsin 53705 USA; ^10^ Department of Inorganic and Analytical Chemistry University of Debrecen Debrecen Hungary; ^11^ Department of Neurosurgery Brigham and Women's Hospital Harvard Medical School Boston MA USA; ^12^ Departments of Chemistry and Molecular Biosciences the Chemistry of Life Processes Institute and the Proteomics Center of Excellence Northwestern University Evanston IL USA; ^13^ Department of Chemistry Aalen University Aalen Germany; ^14^ 908 Devices Inc. Boston MA USA; ^15^ Department of Chemistry and Biochemistry University of Oklahoma Norman OK USA; ^16^ Division of Bioanalytical Chemistry Department of Chemistry and Pharmaceutical Sciences Amsterdam Institute of Molecular and Life Sciences Vrije Universiteit Amsterdam Centre for Analytical Sciences Amsterdam (CASA) Amsterdam The Netherlands

**Keywords:** Analytical methods, Electrophoresis, Mass spectrometry, Proteomics

## Abstract

Mass spectrometry (MS)‐based top–down proteomics (TDP) has emerged as a powerful tool for characterizing proteoforms to advance both fundamental and translational research. TDP requires high‐efficiency liquid‐phase separation, high‐resolution MS, and tandem MS. Capillary zone electrophoresis (CZE)‐MS has been proposed as a promising analytical technique for protein analysis decades ago because of its unique and valuable features, including high separation efficiency and high detection sensitivity. However, CZE‐MS has not been widely adopted by the proteomics community, mainly due to concerns with its robustness and reproducibility. Here, we hypothesized that CZE‐MS is sufficiently robust and reproducible for broad adoption due to the continued efforts of the community over the last three decades. In this work, for the first time, research teams from around the world validated the robustness, repeatability, and reproducibility of CZE‐MS for TDP in both simple and complex model proteoform mixtures employing a full spectrum of commercially available capillary electrophoresis (CE)‐MS interfaces, instrumentation, and compared CZE‐MS performance with state‐of‐the‐art liquid chromatography (LC)‐MS methods. This study offers the research community an informative resource of ready‐to‐use experimental CE‐MS techniques and a better understanding of the CZE‐MS approach and its potential in TDP, accelerating the broad adoption of CZE‐MS in proteoform research.

## Graphical abstract

Mass spectrometry (MS) and top–down proteomics (TDP) have emerged as powerful tools to decipher proteoform‐level information, advancing biological insight and translational research. This study unites leaders in capillary electrophoresis (CE) and MS from multiple institutions across the globe to showcase the reproducibility, unique information, and predictability of CE‐MS for TDP analysis for a diverse set of platforms.

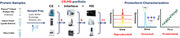

## Introduction

Proteoforms are protein products derived from a single gene that can become functionally distinct due to mutations, polymorphisms, alternative splicing, and post‐translational modifications (PTMs).^[^ ^1^ ^]^ Understanding and characterizing these proteoforms is fundamental in unraveling their roles in biological processes and, thus, advancing our comprehension of human health and disease.^[^ ^2^, ^3^, ^4^ ^]^ While the experimental intact mass of a protein obtained through single‐stage mass spectrometry (MS) can often suggest a specific proteoform,^[^ ^5^ ^]^ particularly with high mass accuracy,^[^ ^6^ ^]^ deeper analysis is typically essential to characterize and identify proteoforms. Identification of the precise modification site, often acting as the definite marker for a proteoform, usually requires tandem mass spectrometry (MS/MS).^[^ ^7^, ^8^, ^9^ ^]^ The analysis of intact proteoforms followed by fragmentation, referred to as “top–down” MS, has been significantly improved over the last two decades, primarily due to advancements in instrumentation as well as putting high emphasis on software development.^[^ ^10^, ^11^ ^]^ Protocols in top‐down proteomics (TDP) distinguish themselves from conventional bottom‐up proteomics approaches by omitting steps that include endoproteinase digestion prior to the analysis.^[^ ^12^ ^]^ This allows the unequivocal characterization of individual intact proteoforms and serves as a complementary method to other MS‐based analyses to enable the analysis of complex proteomes of biological samples.

Primarily, reversed‐phase liquid chromatography (RPLC) has been extensively utilized in TDP as an upfront separation technique due to its well‐established column and stationary phase technologies, compatibility with ESI, and robustness across various HPLC instrumentation and research laboratories.^[^ ^10^, ^11^, ^12^, ^13^, ^14^, ^15^ ^]^ Nonetheless, RPLC encounters challenges in efficiently separating larger intact proteoforms (>30 kDa) due to their strong interactions with the stationary phase, which is typically composed of alkyl‐functionalized porous silica‐based beads. Moreover, mass transfer processes within the stationary phase and from the mobile phase into the stationary phase are particularly slow for large molecular species, leading to substantial band broadening. Recent studies have demonstrated the effective application of ion exchange chromatography as an alternative LC mode of separation in characterizing proteoforms of intact biopharmaceutical proteins.^[^ ^16^, ^17^ ^]^ Complex proteomes, such as those found in human cell lysates, present a serious challenge, estimated to encompass over a million different proteoforms.^[^ ^18^, ^19^ ^]^ To overcome the limitations of conventional techniques (e.g., RPLC) and address current challenges of the proteome complexity, the development of alternative separation techniques with increased efficiency and capacity for proteoform analysis is urgently needed to propel TDP and expedite the progress of the Human Proteoform Project.^[^ ^18^, ^19^, ^20^, ^21^ ^]^

Capillary zone electrophoresis (CZE) emerged in the 1980s as an invaluable technique for separating small and large biomolecules, particularly proteoforms.^[^ ^22^, ^23^ ^]^ CZE is carried out in an open tubular fused‐silica capillary or a channel in a microfluidic device filled with a background electrolyte (BGE) solution, without the need for a stationary phase, under the influence of an electric field. The separation of proteoforms is driven by differences in their electrophoretic mobility (µ_ep_), which is proportional to the ratio of a proteoform's net charge to its hydrodynamic radius. While capillary electrophoresis (CE) is an umbrella term encompassing various capillary‐based electrophoretic techniques, in this paper, we will use the terms CE and CZE interchangeably for the sake of simplicity. Generally, proteoforms exhibit much lower diffusion coefficients compared to peptides and metabolites, leading to exceptionally high separation efficiency of proteoforms (nearly one million theoretical plates) with subtle structural differences, especially if charge‐inducing PTMs are involved.^[^ ^23^, ^24^ ^]^ Furthermore, minimal sample consumption (typically few nL per injection, ng to pg in protein mass), low flow rates (nL min^−1^), and high peak capacity in CE are advantageous for intact proteoform analysis.^[^ ^24^, ^25^, ^26^, ^27^ ^]^

The progress and advantages of CE‐based analysis raise the question of why CZE‐MS has not been more widely deployed in TDP until recently. There is a general notion in parts of the proteomics community that CZE‐MS is neither robust/reproducible nor sensitive enough for efficient proteoform characterization. In addition, CE‐MS interfacing is considered technically challenging compared to LC‐MS, i.e., due to the necessity to apply an electric field across the separation capillary. These concerns have certainly been true historically but have been the focus of industrial and academic development and technological advancements over the last decades.^[^ ^22^ ^]^ For instance, CE‐MS interfacing has been improved dramatically in regard to sensitivity and robustness. Multiple distinct interface iterations, including sheath‐flow and sheathless designs, have been commercialized, making CE‐MS substantially more user‐friendly and accessible.^[^ ^28^, ^29^, ^30^, ^31^, ^32^, ^33^, ^34^, ^35^ ^]^ In addition, different types of CE platforms, including capillary‐ and microfluidic chip‐based, have been established over the past years.^[^ ^29^, ^30^, ^31^ ^]^ These more advanced setups have been successfully employed for characterizing biopharmaceuticals (e.g., monoclonal antibodies, bi‐specific therapeutics, etc.),^[^ ^13^, ^36^, ^37^, ^38^, ^39^ ^]^ profiling proteoforms in cells or tissues globally,^[^ ^40^, ^41^, ^42^, ^43^ ^]^ analyzing single cells,^[^ ^26^, ^30^, ^44^, ^45^, ^46^, ^47^, ^48^ ^]^ understanding critical proteins implicated in disease^[^ ^7^, ^49^, ^50^ ^]^ in a proteoform‐specific manner, deciphering nanoparticle protein corona in nanomedicine,^[^ ^51^, ^52^ ^]^ and enabling native proteomics of endogenous protein complexes in cells.^[^ ^53^, ^54^, ^55^, ^56^, ^57^ ^]^ The separation mechanisms of CZE and conventional RPLC can be viewed as largely complementary (charge‐to‐size ratio versus hydrophobicity). Therefore, CZE‐MS analysis offers additional insights into the proteoform landscape. This was recently demonstrated for the characterization of three human tissues, namely heart, small intestine, and spleen, where samples were analyzed by both RPLC‐ and CZE‐MS‐based TDP workflows.^[^ ^41^ ^]^ In this study, 28% of proteins and 56% of proteoforms were uniquely identified by CZE‐MS, highlighting the gain and complementarity of CZE‐MS‐ and LC‐MS‐based workflows offer to the proteomics community.

**Figure 1:**
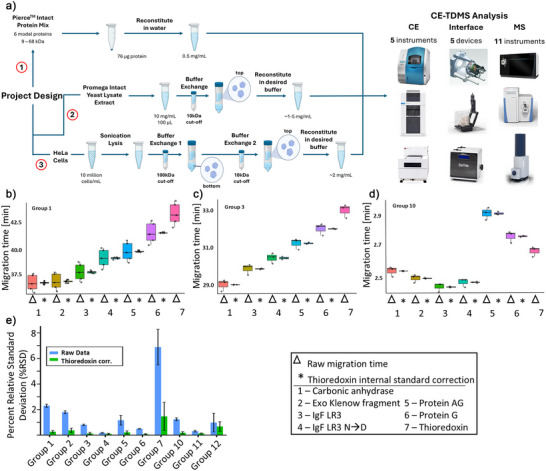
a) General overview of the workflow of the project, with three main experimental arms: the analysis of the Pierce^TM^ intact protein mix, the Promega intact yeast lysate extract, and the analysis of HeLa cell lysate. Representative boxplots of migration times generated by Groups 1, 3, and 10 (Figure 1b–d, respectively) without migration time normalization (Δ) and with normalization using thioredoxin (Trx) as an internal standard (_*_). (e) Bar chart comparing %RSD values before and after internal standard correction (*n* = 3). The error bars in (e) represent the standard deviation of RSD values for different proteins in the sample.

Given recent advances, it is the right time to reevaluate the performance and potential of CZE‐MS for TDP and, in this way, attract more attention and interest to the CE‐MS‐based TDP techniques by presenting the results of this worldwide cross‐laboratory study (12 research groups). The experimental design is shown in Figure 1a. The location distribution of the 12 research groups is shown in Figure S1. Our goal is to provide a repository of information generated in multiple laboratories using a variety of CZE‐MS platforms handled by novices and experts in the field, including capillary‐ and microfluidic chip‐based CE setups (Figure 1a). Additionally, the diversity of CE‐MS interfaces (*n* = 5) and MS platforms (*n* = 11) employed by the participants in this study covers a wide range (i.e., nearly all) of commercially available CE systems and the majority of mass spectrometers suitable for TDP applications, which represents a major novum for such studies. The details of the CE‐MS systems used by the 12 research teams are listed in Table 1. The goal of this study is not to replicate a standardized CE‐MS setup across multiple sites, but rather to showcase the diversity of CE‐MS systems accessible to the TDP community and to demonstrate that reproducible and sensitive results can be achieved with any of these configurations. We also aimed to test the hypothesis that through the continued efforts of the CE‐MS community over the past three decades, CZE‐MS has achieved levels of robustness and reproducibility for broad adoption in the field of TDP, and that it can offer unique, complementary information compared to traditional LC‐based approaches. Optimized methods and benchmark data at this breadth of the evaluated instrumentation will enable users of all skill levels to obtain high‐quality data for the analysis of intact proteoforms with their respective CZE‐MS platforms. Here, we present methods, data analysis approaches, and results acquired for identical samples by nearly a dozen research laboratories around the globe, demonstrating that CZE‐MS is a flexible, powerful, and reproducible technique for intact proteoform analysis in various laboratory settings. In this study, we provide CZE‐MS practitioners with a guide to the best practices at each step of the intact CZE‐MS proteoform analysis workflows and review corresponding benchmark data acquired using representative experimental approaches. Finally, we explore potential future directions for subsequent studies, along with addressing the remaining challenges that still impact CE‐MS in TDP analysis. We will also highlight strategies to overcome these obstacles, aiming to encourage the growth of CE‐MS‐based applications in TDP and potentially other research fields. All method details are described in Supporting Information I.

**Table 1:** The details of CE‐MS systems (CE‐MS interfaces and mass spectrometers) used by the 12 research groups in this study.^a)^

Group	Interface Type	Interface Model	Interface Manufacturer	MS Type	MS Model
1	Sheathless	Sciex OptiMS	Sciex	Orbitrap	Eclipse ‐ Tribrid
2	Nano Sheath‐flow	EMASS‐II	CMP Scientific	Orbitrap	Q Exactive HF
3	Sheathless	Sciex OptiMS	Sciex	Orbitrap	Q Exactive UHMR
4	Sheathless	Sciex OptiMS	Sciex	QTOF	Bruker Impact
5	Sheath‐flow	Agilent Triple‐Tube	Agilent	QTOF	Bruker maXis II
6	Nano Sheath‐flow	nanoCEasy		Orbitrap	Fusion Lumos ‐ Tribrid
7	Nano Sheath‐flow	EMASS‐II	CMP Scientific	TOF	Agilent TOF 6230
8	Nano Sheath‐flow	EMASS‐II	CMP Scientific	Orbitrap	Q Exactive HFX
9	Sheath‐flow	Agilent Triple‐Tube	Agilent	IM‐QTOF	Agilent 6560 IM Q‐TOF
10	Microchip	ZipChip	908 Devices	Orbitrap	Exploris 240 BioPharma
11	Microchip	ZipChip	908 Devices	TOF	Bruker timsTOF Pro
12	Sheathless	Custom		Orbitrap	Exploris 240

^a)^ The Sciex OptiMS interface (Sciex) was developed by the Moini group.^[^ ^34^ ^]^ The EMASS‐II interface (CMP Scientific) was developed by the Dovichi group, and its original name is the electrokinetically pumped sheath‐flow nanospray interface.^[^ ^29^ ^]^ The nanoCEasy is the modified version of the electrokinetically pumped sheath‐flow nanospray interface.^[^ ^31^ ^]^

## Results and Discussion

### CE‐MS Analysis of Intact Protein Standard Mixture

The selected commercially available standard mixture contains six proteins—*Bos taurus* carbonic anhydrase II (CA‐II), insulin‐like growth factor 1 long R3 (IgF‐1 LR3), *Staphylococcus aureus* protein AG (chimeric), *Streptococcus dysgalactiae* protein G, thioredoxin (Trx), and *Escherichia coli* exonuclease (Exo) Klenow fragment (EKFr)—ranging from approximately 9 to 68 kDa (Table S1). According to the manufacturer, IgF‐1 LR3 includes a prominent deamidation variant, bringing the total to seven major analyte species in the sample. This protein standard mix is widely used in protein characterization as a model system for method development, serving as a standard protein ladder in gel electrophoresis. Consequently, it was selected for initial benchmarking and showcase of CE‐MS performance. Moreover, the deamidated variant of IgF‐1 LR3 is particularly valuable for demonstrating the ability of CZE to efficiently separate proteoforms carrying net charge‐modifying PTMs. Twelve research groups analyzed this sample using the CE‐MS instrumentation, protocols, and settings outlined in Protocol 1a and Protocol 1b in Supporting Information I. Detailed experimental conditions are listed in Tables S2 and S3.

Most participants detected at least six of the seven protein species (Table S4). Importantly, ten groups detected and achieved baseline separation of the deamidated IgF‐1 LR3 proteoform, highlighting the ability of CE to resolve and analyze charge variant species—a critical feature in both biological research and biopharmaceutical applications for the regulation and characterization of biological therapeutics. However, only six groups detected EKFr, likely due to its large size (∼68 kDa), and associated challenges for some MS platforms, along with its co‐migration with CA‐II, potentially causing substantial ionization suppression.

The migration order for the observed proteins shows a high level of consistency between research groups using similar CE conditions (Table S4). Variations in BGE composition, particularly increased acid strength or concentration, can impact migration order by shifting pH‐dependent protein charge states and potentially affecting conformation, both key factors in CZE's charge‐ and hydrodynamic volume (i.e., size)‐based separation mechanism. Groups that used relatively similar CE conditions, i.e., Groups 1, 2, 3, and 9, show an extremely consistent migration order: CA‐II migrating first, followed by EKFr (if observed), IgF‐1 LR3, deamidated IgF‐1 LR3, protein AG, protein G, and finally Trx. The one major deviation in terms of migration order was observed in one group using a microchip‐based CE platform with 50% acetonitrile in the BGE, which is expected to modify the migration order relative to aqueous systems. Additionally, the capillary/channel surface chemistry and charge under the conditions of the BGE are important factors that contribute to the observed migration patterns. The average relative standard deviations (RSDs) of migration times for each observed protein across triplicate measurements are less than 3% for eleven labs and about 7% for one lab, indicating reasonable reproducibility of various CE‐MS systems (Table S5). Migration time correction using one of the proteins, Trx, as a proxy internal standard further improved the migration time RSDs to below 1% (except Group 7), (Table S6 and Figure 1b–e), indicating excellent reproducibility of CZE‐MS for protein analysis. Unsupervised hierarchical clustering analysis of Trx‐corrected migration times confirmed that groups using comparable CE‐MS instrumentation and conditions produce similar results. Clustering patterns corresponded with capillary coating chemistries (e.g., linear polyacrylamide used by Groups 1–4; Table S2) and MS optimization, with groups detecting EKFr clustering together (Figure S2). These findings collectively demonstrate strong consistency and reproducibility among CE–MS systems across laboratories.

Notably, the microchip‐based CE system achieved efficient separation and detection of all seven proteoform species in the standard protein mixture in under three minutes, using a field strength of 500 V cm^−1^ (Figure 1d). This is substantially higher than the field strengths used by groups with traditional CE systems (Groups 1–9, 165‐385 V cm^−1^). Such microchip CE systems are well‐suited for high‐throughput applications, including quality control or research and development environments in the biopharmaceutical industry. Overall, all groups achieved separation and detection of observed species in under 45 min, which is at least comparable, if not faster than, the analysis times obtained in traditional reversed‐phase and ion‐exchange LC. Additionally, capillary length, BGE composition, capillary surface coating, and other conditions can be further optimized to meet specific analytical needs, such as an increase in throughput for industrial, academic, or clinical applications. In general, CE systems offer arguably more flexibility than conventional LC approaches as they do not require a specified stationary phase, allowing for more straightforward adjustments and optimization of separation conditions.

**Figure 2:**
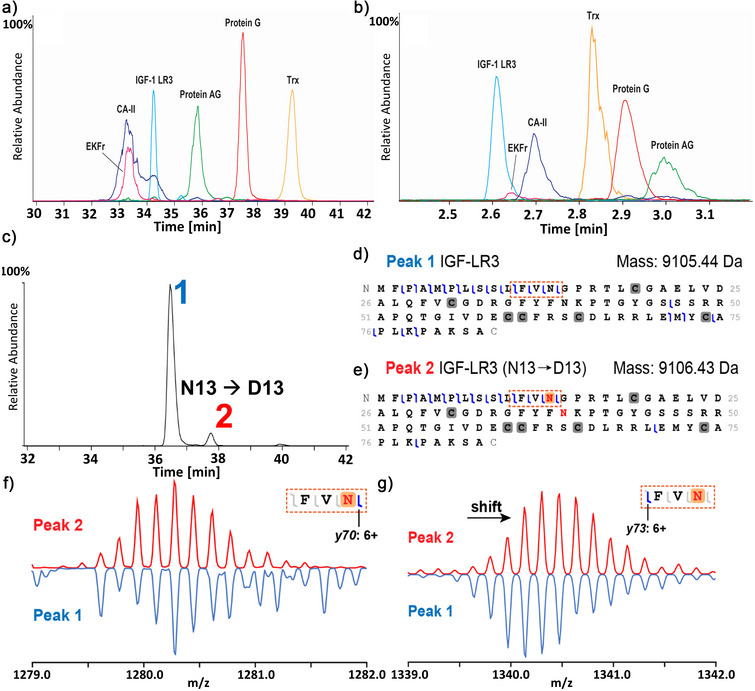
Comparison of representative electropherograms obtained for standard protein mix under denaturing conditions via traditional standard CE instrumentation from Group 1 a) and microchip‐based from Group 10 b). c) Extracted ion electropherogram (EIE) of IGF‐1 LR3 displaying two distinct peaks. EIE trace was generated using the three most abundant charge states. d) and e) Illustrated fragment maps produced by peaks 1 and 2, respectively. Regions of interest for localizing deamidation are outlined by boxes with orange dashed lines. Deconvoluted mass of peak 2 exhibits a distinct mass shift associated with deamidation. f) and g) Comparison of isotopic distributions of diagnostic fragment ions *y70* and *y73* for peak 1 (blue, upside down) and peak 2 (red). A mass shift between peaks 1 and 2 is evident for fragment ion *y73* but not for *y70*, indicating the presence of deamidation at amino acid residue N13.

MS/MS fragmentation was performed by several groups to gather sequence‐level information and PTMs of the proteins (Figures 2 and S3). As mentioned above, one of the distinct features of CE is the ability to efficiently resolve proteoforms containing PTMs that induce a change in the net charge of the protein; for instance, deamidation is a known critical PTM (which may also be a degradation product during biomanufacturing and storage) that adds an additional negative charge to the protein through the conversion of glutamine or asparagine into their acidic analogs. This modification is highly relevant in the production of biotherapeutics as a critical quality attribute (CQA) while also playing an essential role in human health and disease. Figure 2 illustrates the capability to characterize deamidated proteoforms of IgF‐1 LR3 via CE‐MS. Baseline separation between peak 1 and peak 2 was achieved (Resolution = 3.6, *n* = 5; Figure 2c). These peaks exhibit a distinct mass shift (Δ*M* = 0.99 Da), indicative of a deamidation event. Fragment maps for peaks 1 and 2 are depicted in Figure 2d,e. The sequence coverage of the fragment maps was compromised due to the presence of three intact disulfide bridges prior to fragmentation, resulting in limited coverage in the middle part of the amino acid sequence. Nevertheless, clear indicators for the localization of the deamidation sites were observed. The corresponding +1 Da mass shift was detected in all *y*‐ions toward the N‐terminus from the amino acid residue N13 for peak 2, as exemplified by y73 (6+) in Figure 2g. In contrast, fragment ion y70 (6+, between N13 and G14) no longer exhibits the same mass shift (Figure 2f), pinpointing the deamidation site at N13. This precise localization underscores CE's capability to resolve and identify proteoforms (e.g., deamidated and non‐deamidated species) that would co‐elute under reversed‐phase LC conditions, highlighting its exceptional sensitivity to charge‐based PTMs in top‐down proteomics.

### CE‐MS Analysis of Yeast Cell Lysate

**Figure 3:**
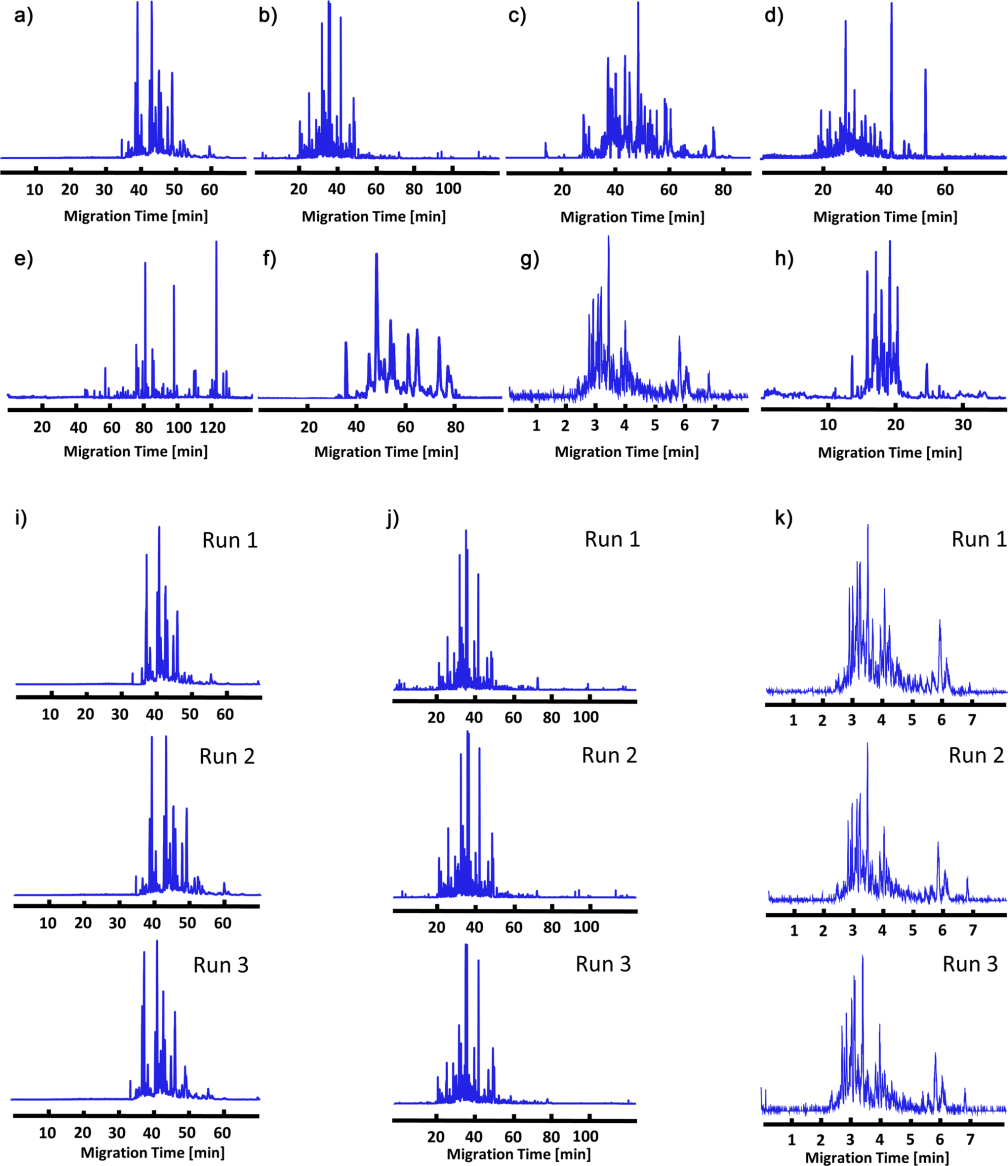
Electropherograms of yeast proteins from eight participants, Groups 1–3, 5–7, 11, and 12, respectively. a)–f) and h) Base peak electropherograms for participants using traditional capillary electrophoresis. g) Base peak electropherogram data using the microchip‐based system. i) Triplicate electropherograms from group 1. j) Triplicate electropherograms from group 2. k) Triplicate electropherograms from group 11.

The intact yeast (*Saccharomyces cerevisiae*) protein extract was analyzed using different settings outlined in Protocol 3, Tables S7 and S8. Figure 3 shows base peak electropherograms for the yeast cell lysate sample obtained by eight participants using various CE‐MS platforms. The different capillary coatings [LPA (Groups 1 and 2), bare fused silica without coating (Groups 3 and 5), PEO (Group 6), PVA (Group 7), PEI (Group 12)] and CE separation channel format [capillary (Groups 1–3, 5–7, and 12) and microchip (Group 11)] can have substantial effect on the CZE separation profile due to changes in electroosmotic flow and interactions of proteins with the capillary surface (Figure 3a–h). Despite these methodological differences, the CZE‐MS separation profiles are highly consistent across the triplicate measurements within each laboratory (Figure 3i–k). Notably, the microchip‐based CZE‐MS system employed a much shorter separation channel (22 cm versus ≥70 cm for capillary systems), allowing high‐throughput analysis of complex samples (Figure 3g,k).

**Figure 4:**
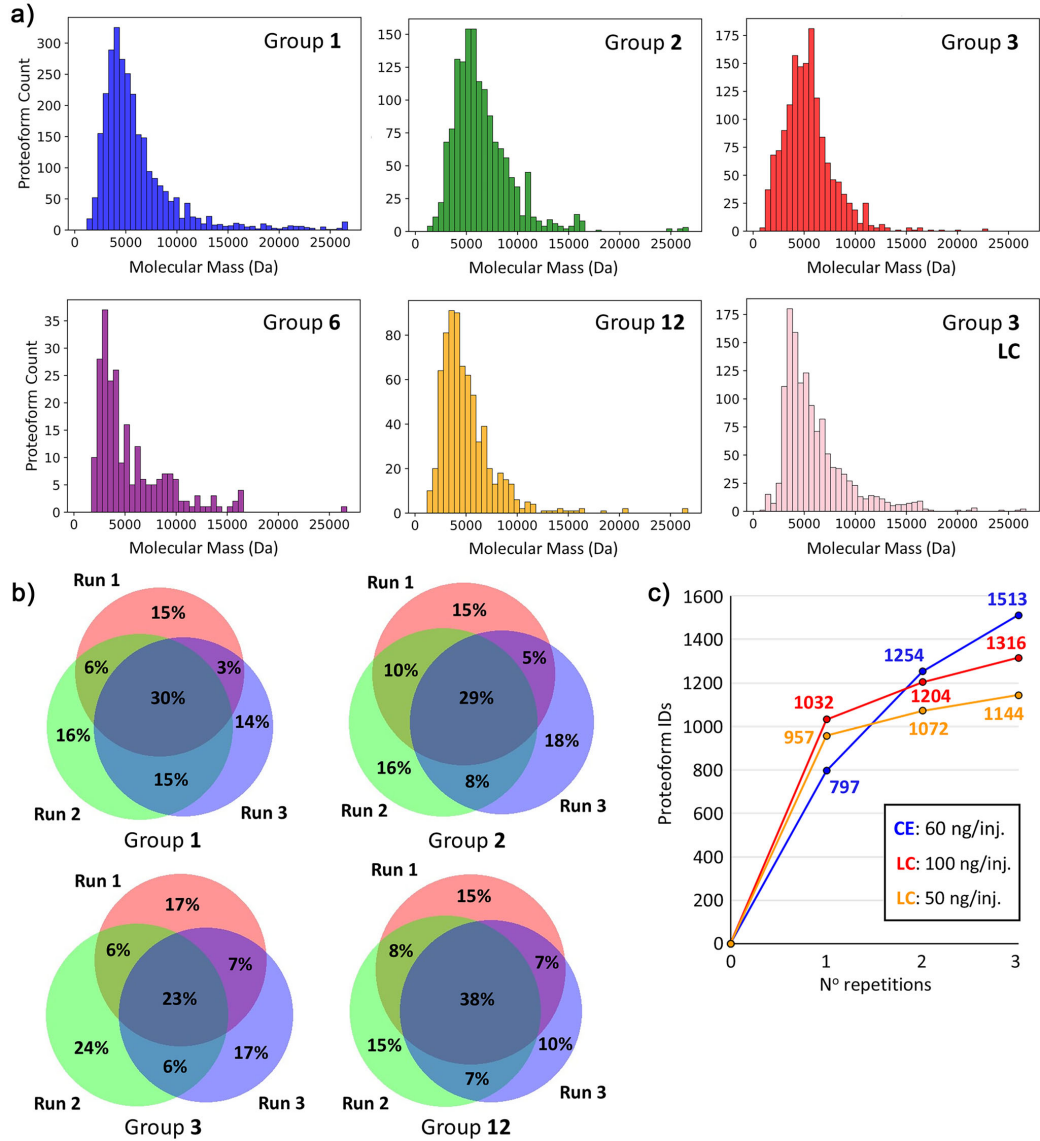
a) Molecular mass distributions of the identified proteoforms from five groups of CE participants and LC data provided by Group 3. b) Venn diagrams for the overlap of identified proteoforms across triplicate runs for the analysis of the yeast cell lysate sample from four study participants. Participants (Groups 1 & 2) used traditional CE instrumentation with near‐neutral linear polyacrylamide‐coated capillaries. Study participants (Group 3) used traditional CE instrumentation with an uncoated, bare‐fused silica capillary. Participants (Group 12) used an in‐house built CE instrument with a positively coated, polyethylenimine, capillary. In all Venn diagrams the red circle, green circle, and purple circle correspond to the first, second, and third runs, respectively. c) Comparison of the observed gains in unique proteoform identifications with increasing replicates for CE (blue) and LC (red and orange).

After database search of raw files containing MS/MS data using the TopPIC Suite pipeline,^[^ ^58^, ^59^ ^]^ between 120–1700 proteoforms corresponding to 50–275 proteoform families were identified from five research groups (Table S9). The higher proteoform counts observed for some groups can be attributed to greater sample injection amounts (18 versus 160 ng), wider CZE separation window (20 versus 60 min), or faster data acquisition duty cycle and sensitivity from more advanced mass spectrometers (e.g., Eclipse Tribrid versus Q‐Exactive HF) (Table S9). Importantly, the number of proteoform identifications was consistent across the triplicate analyses within most laboratories, as reflected by standard deviations below 10% (Table S9). These results demonstrate that reproducible CZE–MS analysis of complex proteomes can be achieved using a range of CE–MS interfaces, instruments, and mass spectrometers. In addition, the molecular mass distributions of identified proteoforms were broadly consistent across research groups (Figure 4a). Group 3 also analyzed the same yeast lysate sample using both CZE‐MS and LC‐MS coupled to the same MS platform under nearly identical conditions. Across groups, the pI distributions of observed proteoforms were similar regardless of capillary coating chemistry (Figure S4). An exception was Group 6, which injected the smallest sample amount (∼20 ng) and detected the fewest number of proteins; the low sample injection amount and number of protein IDs are a probable cause of the slightly altered pI distribution. In Group 3′s comparison, LC–MS yielded a higher proportion of high‐pI proteins than CZE–MS. In the CZE–MS experiment, a 60‐ng injection and 90‐min separation were used, while in LC–MS, a 100‐ng injection and 60‐min effective separation gradient were employed. Despite these differences, both approaches produced comparable proteoform and protein identifications and exhibited similar molecular mass distributions (Figure 4a).

To further assess the reproducibility of proteoform identification across the evaluated experimental conditions, Venn diagrams were generated to compare triplicate yeast lysate analyses from four study participants (Figure 4b). Despite differences in capillary coating, instrumentation, and methods, an average of 30% of proteoforms were consistently identified across all three runs within each group, while only 17% of proteoforms were uniquely identified in a single run, underscoring the method's reliability. Furthermore, Group 3 directly compared LC‐MS (50‐ng injection) and CE‐MS (60‐ng injection) analyses performed on the same mass spectrometer using identical data acquisition parameters (Figure S5). Combined, the two methods yielded 2169 identified proteoforms, with CE‐MS identifying 369 (32%) more proteoforms than LC‐MS at comparable sample loads. Even when the LC‐MS injection amount was doubled to 100 ng, CE‐MS still identified 197 (15%) more proteoforms (Figure 4c). These results highlight the orthogonality and complementarity of the two methods: 48% of the proteoforms were unique to CE‐MS, while only 21% overlapped between the two approaches. In single‐injection experiments, LC‐MS identified more proteoforms than CE‐MS, likely due to longer LC gradients and broader peaks, which increase the number of proteoforms sampled per run. In contrast, CE separations typically produce narrower peaks, which—although generally advantageous—can challenge the MS and MS/MS acquisition rates and duty cycles, potentially limiting identifications per injection. However, the number of unique identifications with CE–MS increased more rapidly with repeated injections, ultimately surpassing LC–MS in replicate injection workflows (Figure 4c). Accordingly, the proteoform identification overlaps across triplicate RPLC‐MS/MS analyses (100 ng injection) was higher than for CZE‐MS/MS (60 ng injection) (Figure S5). Interestingly, RPLC‐MS/MS with 50‐ng injections produced comparable proteoform overlap across triplicate runs to CZE‐MS/MS, likely because the lower LC sample load reduced proteoform signal intensity, leading to more stochastic data acquisition. We also compared yeast protein identifications across CE‐MS datasets from different groups. Among the groups with comparable injection amounts (groups 1, 2, and 3), a total of 359 proteins were observed, with 109 (∼30%) shared across all three groups (Figure S6A). A large portion of the variability in identified proteoforms between groups can likely be attributed to differences in MS methods and instrumentation. When all groups were included, mapping their identified proteins onto a protein copy number per cell S‐curve of the *S. cerevisiae* proteome (Figure S6B) revealed a high degree of overlap among highly abundant proteins.

**Figure 5:**
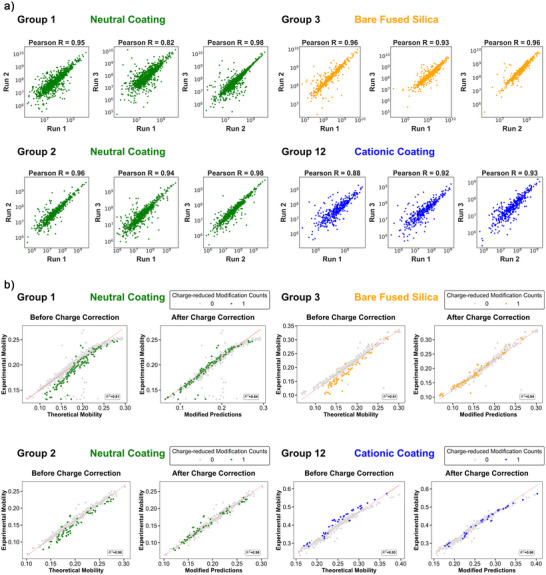
a) The log–log plots of proteoform intensity correlations between triplicates within groups. The Pearson's R score of the linear correlation and capillary condition are listed above each plot. b) The linear correlations between theoretical and experimental electrophoretic mobility (*µ_ep_*) of proteoforms before and after charge correction (see Equation 1). Proteoforms with unidentified mass shifts and proteoforms with migration time less than 12 min were removed for better confidence. Unmodified proteoforms were labeled grey, and proteoforms with varying numbers of charge‐reduced PTMs (acetylation) were labeled in colors specified in inserts. The theoretical electrophoretic mobility is calculated based on the number of positive residues (Ks, Rs, Hs, and the N‐terminal amino group). The charge correction subtracts 1 from the calculated net charge value due to the presence of each charge reducing PTM.

We further evaluated the quantitative reproducibility of CZE‐MS/MS under various instrument conditions using the feature intensity of the overlapped proteoforms between replicates. The correlated intensities of identified proteoforms across triplicates for individual groups are shown in Figure 5a. The Pearson's correlation coefficient values were 0.93 ± 0.05 for all pairwise comparisons, indicating high quantitative reproducibility. Separation consistency was evaluated by comparing migration times of proteoforms observed across runs (Figure S7). For proteoforms detected in multiple runs, migration times plotted against each other showed strong linear correlations (*R* ^2^ > 0.98), demonstrating excellent run‐to‐run reproducibility. Additionally, migration time correction was performed for the runs of Group 12 (Figure S8), which resulted in an improvement in the linearity of the data (i.e., the slope closer to 1). Comparison between CE (Figures S7A and S7B) and traditional nanoflow LC using a packed C4 column (Figure S7C) revealed that CE can achieve separation time reproducibility comparable to LC. Nevertheless, further studies are needed to confirm the long‐term reproducibility of CZE–MS over extended timeframes. High migration time reproducibility is critical for implementing time‐based peak migration alignment and targeted qualitative and quantitative MS data acquisition, which can enhance identification coverage, data consistency, quantitative accuracy, and sensitivity.

One key advantage of CZE is the predictability of migration time based on the assessed electrophoretic mobility, *µ_ep_*. The separation in CZE is driven mainly by the charge‐to‐size ratio of the analytes, and we could employ accurate prediction to cross‐validate the presence of charge‐modifying PTMs. Based on the definition, the experimental electrophoretic mobility (*µ_ep_*) can be determined by dividing the experimental electrophoretic velocity of the solute by the applied electrical field (E), as

(1) $$\mu_{ep}=\frac{v_{ep}}{E}=\frac{\frac{L}{t_{m}}}{\frac{V}{L}}=\frac{L^{2}}{V*t_{m}}$$

where L is the capillary length in cm, *t_m_* is the migration time in seconds, and *V* is the applied potential in kV.

The theoretical *µ_ep, pred_* is calculated based on the semi‐empirical model.^[^ ^60^ ^]^ In brief, the charge *Q* of the analyte of the proteinaceous nature is determined by the number of positively charged amino acid residues (Ks, Rs, Hs, and the N‐terminal amino group) in the acidic background electrolyte. A mass‐to‐charge ratio for the precursor ion is measured by the mass spectrometer and deconvoluted to the experimental molecular mass (*M*).

(2) $$\mathrm{Predicted}\;\mu_{ep,\;\;\;pred}=5*\frac{\ln(1+0.35Q)}{M^{0.411}}$$

As observed in Figure 5b, non‐modified proteoforms aligned closely with the predicted trendline, while proteoforms carrying charge‐reducing PTMs (i.e., acetylation) deviated from it. Accounting for the loss of a positive charge through acetylation improved correlation with the predicted model, confirming the ability of CZE to detect and validate charge‐modifying PTMs. This additional dimension of proteoforms could not only cross‐validate charge‐reducing PTMs but also help resolve unknown mass shifts influenced by charge effects.

Notably, the variation of the theoretical and experimental *µ_ep_* values can be attributed to the different separation conditions, such as internal pressure (e.g., 0.5 psi) during the separation, while the linear relationship remains unchanged. Adjustments for capillary length or separation voltage could be incorporated through scaling coefficients. For cationic coatings, the equation is modified to account for the reversed electroosmotic flow (EOF), by incorporating *µ_EOF_* and a linear coefficient. Importantly, the linearity is consistent across all conditions, including neutral coating, cationic coating, and uncoated negatively charged bare fused silica. Overall, the excellent correlation between experimental and predicted *µ_ep_* of proteoforms using straightforward calculations makes CZE‐MS/MS an attractive analytical method for TDP of complex proteomes with high confidence.

### CE‐MS of HeLa Cell Lysate

**Figure 6:**
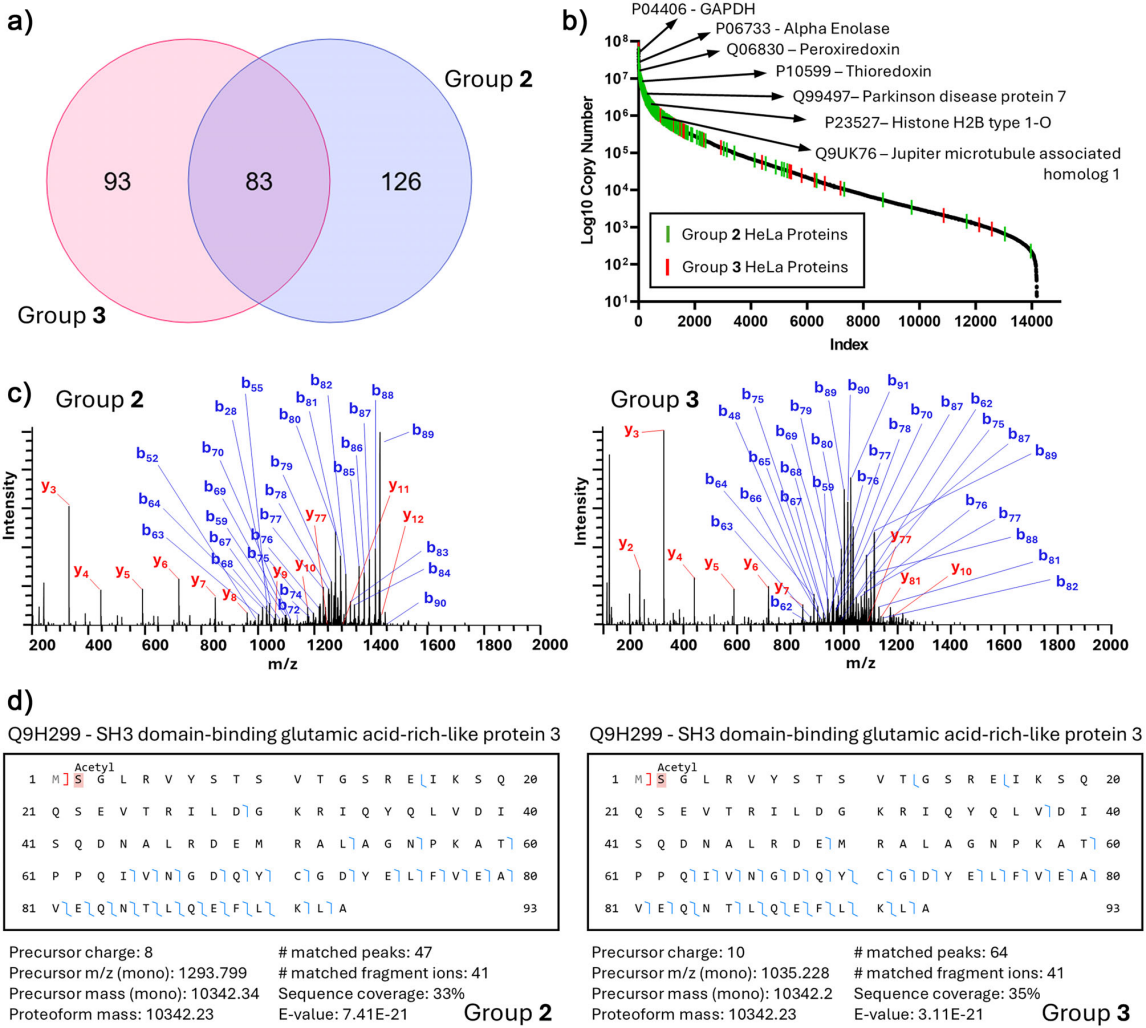
Comparison of CE‐MS top–down proteomic analysis of HeLa lysate between groups 2 and 3. a) Venn diagram of the overlap in observed HeLa proteins between groups 2 and 3. b) S‐curve of protein copy numbers per cell from previously published deep proteomic profiling of HeLa cells, with the observed HeLa proteins from groups 2 and 3 marked with green and red lines, respectively. Select proteins observed across both groups are highlighted. c) Exemplary MS/MS spectrum for SH3 domain‐binding glutamic acid‐rich‐like protein 3 (Q2H299) with identified *b*‐ and *y*‐fragment ions observed by group 2 (left) and group 3 (right). d) Fragmentation map, identification results, and fragmentation coverage for Q2H299 from group 2 (left) and group 3 (right).

To further evaluate the CZE‐MS/MS technique for complex proteomic samples, we analyzed HeLa cell lysates using two independent CZE‐MS/MS systems (Groups 2 and 3). Samples were prepared according to protocol 4 and analyzed using settings outlined in Table S10. Group 2 identified a total of 209 HeLa proteins and 714 proteoforms across three runs with an injection amount of ∼100 ng, while group 3 observed 176 proteins and 441 proteoforms across three runs with an injection of ∼20 ng of protein. Group 2 detected an average of 171 proteins and 495 proteoforms per run with RSDs of 2% and 5%, respectively. Group 3 detected an average of 108 proteins and 219 proteoforms per run with RSDs of 2% and 13%, respectively. 83 proteins (∼27%) were identified by both groups (Figure 6a). Similar to the yeast analysis, we mapped the overlapping HeLa proteins to the reported protein copy numbers and onto a resulting HeLa proteome S‐curve, highlighting seven exemplary proteins observed by both groups that are known to play key roles in cellular function and pathologies (Figure 6b). Additionally, we examined the MS/MS spectra for proteoforms characterized by both groups 2 and 3 and observed comparable levels of fragmentation and sequence coverage. An example is shown in Figure 6c,d, showing the MS/MS spectra and sequence coverage of one SH3 domain‐binding glutamic acid‐rich‐like protein 3 proteoform from both groups. Although slight variations were observed in the distribution of *b*‐ and *y*‐ions between the two groups due to some differences in MS/MS settings (Table S10), the agreement on the overall fragmentation patterns and PTM across two independent CZE‐MS systems demonstrate the consistency of CZE‐MS/MS for proteoform characterization in complex biological samples under well‐controlled conditions.

## Conclusion

In this work, we systematically assessed the performance and consistency of various CZE approaches, CE‐MS interfaces, and MS platforms across multiple laboratories worldwide for TDP characterization of both model protein standard mixtures and complex cell lysates. Using identical samples, we assessed both capillary‐based and microfluidic CE‐MS systems. Our results demonstrated a high degree of migration time reproducibility for intact protein standards, with relative standard deviation (RSD) values below 1%. The diversity of CE instrumentation, ranging from commercial to home‐built systems with various capillary geometries and chemistries, supports industry‐standard throughputs while preserving CE's intrinsic size‐ and charge‐based separation capabilities for TDP analysis. We highlighted CE‐MS’s strength to efficiently resolve proteoforms carrying charge‐altering PTMs, such as achieving baseline separation of a deamidated IgF‐LR3 proteoform from its unmodified counterpart. CE‐MS also proved highly effective for TDP of complex proteomes, yielding more proteoform identifications than traditional nanoLC‐MS‐based TDP using bead‐packed C4 columns with comparable sample loads. The orthogonality between CE‐MS and RPLC‐MS was demonstrated, with almost 50% of total proteoforms being uniquely identified by CE‐MS, underscoring the potential gain CE‐MS provides for the TDP community. Furthermore, analyses of cell lysates demonstrated CE–MS's reproducibility and predictive capability (yeast lysate), as electrophoretic mobilities and migration times closely matched semi‐empirical predictions. We also showed that CE‐MS can be used for TDP of more biologically relevant complex samples, such as immortalized human cell lines (HeLa), and achieve similar levels of identifications and overlapping identifications across different instrumentation and groups as was observed in the analysis of the yeast lysate sample. Collectively, these findings provide strong evidence that CZE‐MS/MS is ready for broad adoption for TDP of complex biological samples.

This study involves the evaluation of both capillary‐ and microfluidic chip‐based CZE separations. Microfluidic CZE‐MS provides clear advantages for high‐throughput analysis, as demonstrated by our data, owing to the short separation channel (i.e., 10 and 22 cm) and strong electric field achievable in the commercial microfluidic CE system. In contrast, capillary‐based CZE‐MS typically requires a substantially longer separation capillary (i.e., ∼60–100 cm) due to the design of commercial CE systems, which limits throughput. However, the use of longer capillaries enables higher peak and sample loading capacity separations of complex proteoform mixtures, thereby enhancing proteome coverage, as supported by our data. Furthermore, as we showcased here, various CE‐MS interfaces can be employed in TDP to achieve reproducible proteoform separations and detection. Each CE‐MS interface has distinct operational characteristics that have been comprehensively summarized in recent reviews.^[^ ^22^ ^]^ We recommend that new users consider these features, along with the specific project goals (i.e., high‐throughput versus deep coverage), when selecting a CZE‐MS platform. Additionally, we encourage participation in practical training opportunities, such as training sessions provided by CE‐MS instrument vendors and the annual CE‐MS summer school organized at Michigan State University.

The participation of multiple CE‐MS experts from laboratories around the globe in this study prompted discussions on current challenges and future directions for advancing CE‐MS in TDP. A major challenge in TDP of complex mixtures, such as total lysates, remains the reduced proteome coverage compared to traditional bottom–up analysis. Difficulties in measuring large proteoforms (i.e., >30 kDa), membrane proteoforms, and heavily modified proteoforms stem from issues related to the analyte solubility, frontend separation performance, ionization efficiency, desolvation/declustering efficiency, resolution of closely spaced ion species, gas‐phase fragmentation efficiency, detection sensitivity, and data analysis. Another potential challenge shared by all nanoflow liquid phase separation‐based techniques (e.g., CE and LC), but especially pronounced in the techniques utilizing pulled nano‐electrospray emitters, is the suboptimal electrospray stability when analyzing samples of increased complexity. Sample constituents, such as lipids, detergents, and cell debris, can negatively impact spray stability and even block the capillary. CE–MS typically employs capillaries with uniform inner diameters or microfluidic channel cross‐sections and unrestricted tips, making them less prone to clogging, provided that effective high‐pressure rinsing is implemented between analyses. While CZE‐MS offers several advantages, it may suffer from its low sample loading capacity for complex samples. Several additional improvements in enhancing sample loading capacity and capillary inner surface coating chemistries will further advance CZE‐MS/MS for TDP of complex biological samples. Recently, significant efforts have been made to boost the sample loading capacity, e.g., dynamic pH junction and solid phase microextraction to better tackle “real‐world” sample volumes, especially in single‐cell and limited sample analysis applications.^[^ ^27^, ^61^, ^62^ ^]^ Additionally, further investigations into long‐term reproducibility across multiple replicates and separation capillaries and microchips, which was beyond the scope of this project, will be instrumental for the research community to fully address concerns about robustness and reproducibility. Recent work has already been reported^[^ ^63^ ^]^ that shows the long‐term reproducibility of CZE‐MS for TDP, and it serves as a helpful template for future studies using different sample types. Finally, the chemistry of the capillary inner wall coating is central for achieving highly efficient proteoform separations, especially for the analysis of large proteoforms. Both neutral and cationic capillary coatings have been broadly employed for CZE‐MS/MS‐based protein and proteoform studies.^[^ ^22^, ^37^, ^64^, ^65^, ^66^, ^67^ ^]^ Continued research into coating reproducibility, stability, and applicability to diverse proteoform classes, e.g., large proteoforms, membrane proteins, and protein complexes, will further enhance the performance and utility of CE‐MS for comprehensive proteoform characterization.

Based on the current state of the field and recent technological advances, we identify several promising and impactful application areas for CE‐MS in TDP: I) CE‐MS‐based TDP appears to be an attractive approach to discovering novel proteoform biomarkers of diseases (e.g., cancer and Alzheimer's disease).^[^ ^4^, ^42^, ^68^ ^]^ II) CE‐MS can be instrumental in the top‐down MS‐based characterization of biopharmaceuticals (e.g., monoclonal antibodies, bi‐/tri‐specific nanobodies, among others) due to its high separation resolution (especially for charge variants and variably modified species) and superb detection sensitivity.^[^ ^13^, ^36^, ^37^, ^69^, ^70^ ^]^ III) CE‐MS also shows great potential for native TDP, which aims to achieve proteome‐wide characterization of protein complexes in cells and other biological systems.^[^ ^53^, ^57^, ^71^, ^72^ ^]^ IV) Targeted native CE‐MS applications are poised to become valuable tools in structural biology, providing detailed insights into protein complex stoichiometry, conformational states, and interactions.^[^ ^54^, ^55^, ^56^ ^]^ V) Due to its minimal sample consumption and high detection sensitivity, CE‐MS offers unique advantages for the analysis of amount‐limited biomedical samples, including single cells and sub‐µL aspirates from scarce biological and clinical sources.^[^ ^26^, ^44^, ^45^, ^46^, ^48^ ^]^ VI) Finally, CE‐MS‐based TDP holds transformative potential for applications in nanomedicine, early‐stage disease diagnostics, and monitoring of therapy treatment outcomes. The proteoform level resolution can be leveraged to characterize nanoparticle coronas, single cells, extracellular vesicles and particles, and proteoform alterations in tissues and biofluids,^[^ ^51^, ^52^, ^73^ ^]^ potentially reshaping our understanding of biology manifested in such sample types and advancing nanomedicine.

A central objective of this study was to demonstrate that CE‐MS possesses the reproducibility and sensitivity required for effective proteoform characterization and that it is suitable for broad adoption in TDP analysis. Across multiple platforms and laboratories, CE‐MS showed strong reproducibility, achieving relative standard deviations (RSDs) below 1% for migration times after internal standard correction. When directly compared with LC‐MS data generated in the same laboratory under identical MS conditions and data analysis parameters, CE‐MS showed comparable sensitivity and produced similar numbers of proteoform identifications across triplicate runs. It is important to note that LC‐MS‐based TDP continues to advance rapidly.^[^ ^74^, ^75^, ^76^ ^]^ Although the LC‐MS performance in this study aligns with previously published results under comparable conditions,^[^ ^74^, ^75^, ^76^ ^]^ it may not fully represent the current, rapidly advancing state‐of‐the‐art in LC‐MS‐based TDP. Nonetheless, the advantage of CE‐MS was pronounced in the large number of uniquely identified proteoforms, nearly 50%, which were undetected by the LC‐MS platform, consistent with previous findings.^[^ ^41^ ^]^ This comparison underscores the complementary nature of these two separation techniques and highlights the value of incorporating CE‐MS into the suite of tools consistently used in proteomics research. Finally, due to the narrow peak shapes produced in CE, some proteoforms may still remain undetected as MS/MS acquisition may struggle to keep pace with the efficiency of CE‐based separation. However, this limitation also represents a significant opportunity. As advances in MS acquisition speed and parallelization continue, CE–MS is poised to capitalize on these developments, enabling even deeper and more comprehensive proteoform coverage in future TDP studies.

## Supporting Information

Experimental methods, data analysis description, and additional supporting tables and figures can be found in the supporting information file. The mass spectrometry proteomics data have been deposited to the ProteomeXchange Consortium via the PRIDE^[^ ^77^ ^]^ partner repository with the dataset identifier PXD059108.

## Author Contributions

N.G. and Q.W. contributed equally by preparing all figures and writing the manuscript with A.R.I., K.J., and L.S. A.R.I., K.J., and L.S. (names of the corresponding authors are listed in alphabetical order) conceived the study design and contributed equally to this project. Experiments were performed by N.G., Q.W., D.C., F.F., M.S.F, MJ.G., M.G., C.G., N.H., A.H., M.A.H., K.J.J., T.K., E.J.L., E.A.R., J.S, Z.Z, and G.Z., who all contributed data that was incorporated into the final results. All authors contributed to the discussions as well as the editing and formatting of the manuscript.

## Materials and Correspondence

Correspondence and material requests should be addressed to Alexander R. Ivanov (a.ivanov@northeastern.edu), Kevin Jooß (k.jooss@vu.nl), and Liangliang Sun (lsun@chemistry.msu.edu).

## Conflict of Interests

Authors with company affiliations are as follows: D.C. (Merck and Co., Inc., South San Francisco, CA, USA), J.S.M (908 Devices Inc.), and E.A.R (908 Devices Inc.). Otherwise, the authors declare there are no known competing financial interests or personal relationships that could have influenced the work reported in this paper.

## Supporting information



Supporting Information

## Data Availability

The data that support the findings of this study are openly available in PRIDE^[^ ^77^ ^]^ at www.ebi.ac.uk/pride/, under the dataset identifier PXD 059108.
