# Supplementary material for: Intact Proteoform Analysis by Capillary Electrophoresis–Mass Spectrometry. Are We There Yet?
Source: Angew Chem Int Ed Engl. 2025 Dec 18;65(5):e18366. doi: 10.1002/anie.202518366 (PMC12814393; doi:10.1002/anie.202518366)
Supplement: Supplementary file 1 — Supporting Information [file ANIE-65-e18366-s001.docx]

**Supporting Information**

**Intact Proteoform Analysis by Capillary Electrophoresis – Mass Spectrometry. Are We There Yet?**

Noah Gould^1,#^, Qianjie Wang^2,#^, Jeffrey N. Agar^1^, Jennifer S. Brodbelt^3^, Daoyang Chen^4^, Kellye A. Cupp-Sutton^5^, Elena Domínguez-Vega^6^, Fei Fang^2^, Marianne Fillet^7^, Matthew S Fischer^8^, Attila Gaspar^10^, Ying Ge^8,9^, Marie-Jia Gou^7^, Michal Greguš^1^, Christoph Gstöttner^6^, Narmin Hamidli^10^, Amanda Helms^3^, Md Amin Hossain^1,11^, Kyle J. Juetten^3^, Neil L. Kelleher^12^, Tobias Kraus^13^, Eli J Larson^8^, J. Scott Mellors^14^, Cynthia Nagy^10^, Christian Neusüß^13^, Erin A. Redman^14^, Jasmin Schairer^13^, Si Wu^5,15^, Tian Xu^12^, Zhitao Zhao^5,15^, Guijie Zhu^2^, Alexander R. Ivanov^1*^, Kevin Jooß^16,17*^, Liangliang Sun^2*^

^1^Barnett Institute of Chemical and Biological Analysis and Department of Chemistry & Chemical Biology, Northeastern University, Boston, MA, USA.

^2^Department of Chemistry, Michigan State University, East Lansing, MI, USA.

^3^Department of Chemistry, University of Texas, Austin, TX, USA.

^4^Discovery Biologics, Merck Research Laboratory.

^5^Department of Chemistry and Biochemistry, University of Alabama, Tuscaloosa, AL, USA.

^6^Center for Proteomics and Metabolomics, Leiden University Medical Center, Leiden, The Netherlands.

^7^Laboratory for the Analysis of Medicines, Center for Interdisciplinary Research on Medicines (CIRM), University of Liege, Quartier Hospital, Liege, Belgium.

^8^Department of Chemistry, University of Wisconsin-Madison, Madison, Wisconsin 53706, United States

^9^Department of Cell and Regenerative Biology and Human Proteomics Program, University of Wisconsin-Madison, Madison, Wisconsin 53705, United States

^10^Department of Inorganic and Analytical Chemistry, University of Debrecen, Debrecen, Hungary.

^11^Department of Neurosurgery, Brigham and Women’s Hospital, Harvard Medical School, Boston, MA, USA.

^12^Departments of Chemistry and Molecular Biosciences, the Chemistry of Life Processes Institute, and the Proteomics Center of Excellence, Northwestern University, Evanston, IL, United States

^13^Department of Chemistry, Aalen University, Aalen, Germany.

^14^908 Devices Inc., Boston, MA, USA

^15^Department of Chemistry and Biochemistry, University of Oklahoma, Norman, OK, USA.

^16^Division of Bioanalytical Chemistry, Department of Chemistry and Pharmaceutical Sciences, Amsterdam Institute of Molecular and Life Sciences, Vrije Universiteit Amsterdam, Amsterdam, The Netherlands.

^17^Centre for Analytical Sciences Amsterdam (CASA), The Netherlands

^#^These authors contributed equally: Noah Gould, Qianjie Wang.

Correspondence to: *email: [a.ivanov@northeastern.edu](mailto:a.ivanov@northeastern.edu), [k.jooss@vu.nl](mailto:k.jooss@vu.nl), [lsun@chemistry.msu.edu](mailto:lsun@chemistry.msu.edu) (equal contribution corresponding authors, in alphabetical order)

**Table of Contents**

Materials and Methods……………………………………………………………………………3

Supplemental Table 1……………………………………………………………………………..8

Supplemental Table 2……………………………………………………………………………..9

Supplemental Table 3……………………………………………………………………………10

Supplemental Table 4…………………………………………………………………………....11

Supplemental Table 5……………………………………………………………………………12

Supplemental Table 6……………………………………………………………………………13

Supplemental Table 7……………………………………………………………………………14

Supplemental Table 8……………………………………………………………………………15

Supplemental Table 9……………………………………………………………………………16

Supplemental Table 10…………………………………………………………………………..17

Supplemental Figure 1…………………………………………………………………………...18

Supplemental Figure 2…………………………………………………………………………...19

Supplemental Figure 3…………………………………………………………………………...20

Supplemental Figure 4…………………………………………………………………………...21

Supplemental Figure 5…………………………………………………………………………...22

Supplemental Figure 6…………………………………………………………………………...23

Supplemental Figure 7…………………………………………………………………………...24

Supplemental Figure 8…………………………………………………………………………...25

References………………………………………………………………………………………..26

**Supporting Materials and Methods**

**Methods**

***Analysis of Intact Protein Standards and Benchmarks***

To supply a standardized sample and a set of experimental conditions with resulting datasets that CE-MS practitioners can use as a starting point and to benchmark their own experiments and method performance, we provide protocols and results for the analysis of commercially available protein samples of various complexity: (i) the Intact Protein Standard Mix (Pierce, PN A33526), (ii) MS Compatible Yeast Protein Extract, Intact (Promega, V7341).

Raw data were collected from each group and put into a central repository, then migration times for individual proteins were determined using the observed migration time window for the protein in its corresponding extracted ion electropherogram. Migration time correction was performed using Trx as a proxy internal standard; in short, observed migration times for individual proteins were expressed as a fraction of the migration time for Trx observed in that run, and then multiplied by the average migration time observed for Trx across the triplicate measurements.

***1. Preparation and Analysis of Intact Protein Standard Mix***

***Protocol 1a: Preparation of Intact Protein Standard Mix***

The Pierce^TM^ mix contains six lyophilized proteins of various sizes (9 – 68 kDa) and was prepared by resuspension in either deionized water or 100 mM ammonium acetate (AmAc) at pH 6.7, to be amenable to potential pH-mediated stacking enrichment, to reach the desired concentration for analysis (*e.g.*, 0.5 mg/mL). After resuspension, the sample was vortexed for 2 minutes before centrifugation at 14,000 xg for 10 minutes at 4 ^o^C, after which it was ready for analysis or to be stored at -80 ^o^C.

***Protocol 1b: Analysis of Intact Protein Standard***

In the first part of this project, all groups used various CE-MS approaches for the analysis of the intact protein standard. With the wide variety of CE instrumentation and interfaces, it is not possible to provide a single, all-encompassing protocol. The availability of CE instrumentation will control many aspects of the CE analysis and separation, such as capillary/channel length, inner and outer diameter (or channel geometry), voltage, and the necessity of supplemental pressure. Additionally, there were differences in capillary/channel inner surface coating and BGE. These aspects could all result in independent effects potentially influencing the separation and analysis of intact proteins. These conditions largely varied from group to group as they were closely related to the available instrumentation and established practices of each research laboratory. Considering this, we analyzed the same intact protein standard across several labs, with a variety of different commercial CE instrumentation and diverse approaches to CE analysis (**Table S2**).

The identification and characterization of intact proteins, even when subjected to denaturing conditions, necessitates a high-resolution mass spectrometer to obtain sufficient mass accuracy. In the field of intact protein MS analysis, the three most utilized mass spectrometers, categorized by the type of mass analyzer integrated into the instrument, are Time-of-flight (TOF), Orbitrap (OT), and Fourier transform-ion cyclotron resonance (FT-ICR) systems. The settings for the MS instrumentation and methodologies varied among participants, and an overview is given in **Table S3**. In summary, the participant groups can be split into two categories: those employing TOF or Orbitrap-based mass spectrometers.

***2. Preparation and Analysis of Intact Yeast Protein Extract***

***Protocol 2: Preparation of Intact Yeast Protein Extract***

Three different sample preparation protocols for the intact MS-compatible yeast protein extract were provided, including: (I) acetone precipitation, (II) ultrafiltration-based buffer exchange, and (III) simple dilution (with and without urea). Each protocol was tested and verified for its validity in a prior experiment. These protocols served as guidance for the study participants and could be adapted to their individual needs accordingly. Initially, the sample was resuspended in 100 µL of Milli-Q water (or equivalent), resulting in a stock sample with a concentration of 10 mg/mL, which could be used for subsequent sample preparation protocols.

***(I) Acetone Precipitation of Intact Yeast Protein Extract***

A 5 µL aliquot of the stock sample (~50 µg) was diluted to 40 µL with 8 M urea in Milli-Q water (or equivalent), followed by the addition of 5 µL of 1 M dithiothreitol (DTT, reduction solution, pH 8) in water. The solution was incubated at 37 ^o^C for 60 minutes. Following reduction, 4× the sample volume (200 µL) of cold acetone (-20 ^o^C) was added, the sample was vortexed and incubated at -80 ^o^C for 60 minutes. Subsequently, the sample was centrifuged for 10 minutes at 14,000×g; after centrifugation, the supernatant was carefully disposed of without disturbing the protein pellet. A 4x the sample volume (200 µL) of cold acetone was added (optional – break up the pellet by pipetting the sample up and down), then centrifuged at 14,000×g for 10 minutes. Next, the supernatant was removed, and the pellet was allowed to dry partially by leaving the tube uncapped at room temperature for 10 minutes. It is important not to completely dry the pellet, since this can cause issues with dissolving the pellet during the next step. Subsequently, 100 mM AmAc (adjusted to pH 8 with ammonia) was added to dissolve the pellet, and a volume suitable for downstream analysis was used.

***(II) Ultrafiltration-based Buffer Exchange***

Initially, stock solutions of (I) 8 M urea and (II) 200 mM AmAc (adjusted to pH 8.0) were prepared. Using these stock solutions, 100 mM AmAc pH 8.0 (dilution of stock solution II in Milli Q water or equivalent) and a 100 mM AmAc: 4 M urea solution (1:1 mixture of stock solutions) were prepared. The 100 AmAc pH 8.0 solution was used to wet a 10 kDa Amicon spin-column (or identical products; 0.5 mL column volume) and centrifuged at 14,000×g for 20 minutes at room temperature. A 5 µL aliquot of the 10 mg/mL stock sample was diluted to 100 µL with the 100 mM AmAc, pH 8.0: 4 M urea solution and added to the spin-column or filter and centrifuged at 14,000×g for 20 minutes at room temperature, which resulted in roughly 30 µL of the sample left in the unit. A volume of 200 µL of 100 mM AmAc was added to the resulting sample on top of the filter membrane and centrifuged at 14,000×g for 25 minutes at 4 ^o^C. This step was repeated three times. The filtration unit was moved to a new Eppendorf tube, vortexed thoroughly, and then the unit was inverted and centrifuged at 3,000×g for three minutes to collect the sample, which was ready for analysis.

***(III) Sample Dilutions (with and without urea)***

A 5 µL aliquot of the 10 mg/mL stock sample was diluted to 50 µL with 100 mM AmAc: 4 M urea, vortexed, and centrifuged for 10 minutes at 14,000×g. Alternatively, 5 µL of the sample stock was diluted to 50 µL using 100 mM AmAc pH 8.0, and followed the same procedure as described above for centrifugation. These preparation protocols are much simpler and faster compared to the acetone precipitation (I) and ultrafiltration-based (II) approaches. However, they are less efficient in removing potential contaminants and salts that can negatively influence subsequent CE-MS analysis.

***Protocol 3: Analysis of Intact Yeast Protein Extract***

We first compared the three sample preparation protocols for the yeast cell lysate in terms of the number of proteoform identifications. The number of proteins and proteoforms identified between the buffer exchange (II) and the dilution approach without urea (III) were comparable, with the buffer exchange protocol delivering slightly higher numbers in IDs. In comparison, a distinct drop in protein/proteoform IDs was observed following the acetone precipitation protocol (I). This could be explained by some proteins not fully redissolving after the precipitation step, which is substantiated by the loss of certain peaks in the electropherogram, while others seem not to be impacted (data not shown). Furthermore, the addition of urea into the sample mixture for the simple dilution protocol (III) significantly reduces the number of identified proteins/proteoforms and, therefore, should be avoided.

Eight of the participating groups have completed the analysis of the yeast protein extract, using multiple different CE instrumentation, CE-MS interfaces, and mass spectrometers. The key conditions for CZE analysis and MS settings are listed in **Tables S7** and **S8**, respectively. Different from the relatively simple intact protein standards, the sample injection volume of the yeast was increased, utilizing pH-mediated stacking [27, 73], to improve the identification of lower abundance proteoforms in the complex mixture.

***Liquid Chromatography Analysis of Yeast Protein Extract***

An Ultimate 3000 nanoLC system (Thermo Fisher Scientific) was used for nanoLC-MS experiments for TDP experiments. A 75 cm, 75 μm ID, 360 μm outer diameter capillary was packed with ReproSil-Pur, C4, 5 μm, 300 Å stationary phase (Dr. Maisch, Ammerbuch, Germany). Sample was loaded at a flow rate of 200 nL/min for 15 minutes with 5% B (acetonitrile, 0.1% formic Acid). Proteins were then eluted from the column at 200 nL/min with a 60 min linear gradient from 5-40% B, where solvent A consisted of 0.1% FA in water and solvent B consisted of 0.1% FA in acetonitrile (ACN). The solvent composition was then stepped to 85% B over 1 minute and then held at 85% B for 3 minutes. Lastly, the solvent composition was changed from 85% B to 5% B over 1 minute and then held constant for 20 minutes to re-equilibrate the column.

***Database Search of Yeast Protein Extract***

The RAW files were analyzed with the TopPIC Suite pipeline [58]. First, the RAW files from all groups were converted into mzML files with the peak picking algorithm provided by MSConvert. Then, spectral deconvolution was performed with TopFD (version 1.5.2) with default parameters, which generates the neutral monoisotopic masses and finds proteoform features by combining precursor isotope clusters with similar monoisotopic masses and close migration times in MS1 scans [59]. The result files for the yeast lysate sample analysis were searched against the concatenated target-decoy database using TopPIC (version 1.5.2). The yeast proteome database was downloaded from UniProt (UP000002311, 6735 entries, version March 24, 2023). The precursor and fragment mass error tolerances were 10 ppm, and the error tolerance for identifying PrSM cluster was 1.2 Da. The maximum number of variable modifications was set to 3. The N-terminal modification allowed four different forms: none, methylation, acetylation, and acetylation after methionine removal. The maximum mass shift of unknown modifications was set to 500 Da. The maximum number of unexpected modifications was two. The proteoforms identified from the replicates were merged and filtered with a 1% spectrum-level FDR and a 5% proteoform-level FDR.

***3. Preparation and Analysis of HeLa Cell Lysate***

***Protocol 4. Preparation of HeLa cell lysate***

***(I) Group 2 preparation of HeLa cell lysate***

The HeLa cells were lysed using a lysis buffer containing 8M urea and 100 mM ammonium bicarbonate plus the complete protease inhibitors and phosphatase inhibitors, with the assistance of sonication on ice using a Branson Sonifier 250 (VWR Scientific, Batavia, IL). After centrifugation, the proteoforms in the supernatant were reduced by dithiothreitol (DTT). After that, an aliquot of the HeLa proteoform sample containing about 1 mg proteins was transferred onto a 100-kDa molecular weight cut-off (MWCO) centrifugal filter unit to remove the large proteoforms and collect small proteoforms in the flow-through after centrifugation. The collected small proteoforms were buffer-exchanged into an ammonium acetate buffer using a 10-kDa MWCO centrifugal filter unit, followed by CZE-MS/MS analysis.

***(II) Group 3 preparation of HeLa cell lysate***

HeLa cells were cultured individually by groups 2 and 3 (see supplemental information). HeLa cells were lysed and buffer exchanged using the following protocol. HeLa cells were lysed using sonication, and then the cell debris was removed from the sample by centrifugation for 30 minutes at 10,000 RPM. The supernatant was then collected and filtered through a 100 kDa Amicon spin-column to remove large complexes, aggregates, and leftover cell debris, and the flow-through collected for further filtering. The flow-through was then buffer exchanged and filtered using a 10 kDa Amicon spin-column, in order to remove excess salts and exchange the sample into a buffer suited for CE-MS analysis, after which the column was inverted, placed into an Eppendorf tube, and centrifuged at 3,000 x g for 3 minutes to collect the sample.

***Database Search of HeLa Cell Lysate***

The RAW files from both CE-MS- and LC-MS-based experiments were analyzed with the TopPIC Suite pipeline [58]. First, the RAW files were converted into mzML files using the peak picking algorithm by MSConvert. Then, spectral deconvolution was performed with TopFD (version 1.5.2) with default parameters, which generates the neutral monoisotopic masses and finds proteoform features by combining precursor isotope clusters with similar monoisotopic masses and close migration times in MS1 scans [59]. The UniProtKB/Swiss-Prot human database (Release 2021_04, containing 20,375 reviewed sequences) was used for the TopPIC search. The precursor and fragment mass error tolerances were 10 ppm, and the error tolerance for identifying PrSM cluster was 1.2 Da. The maximum number of variable modifications was set to 3. The N-terminal modification allowed four different forms: none, methylation, acetylation, and acetylation after methionine removal. The maximum mass shift of unknown modifications was set to 500 Da. The maximum number of unexpected modifications was two. The proteoforms identified from the replicates were merged and filtered with a 1% spectrum-level FDR and a 5% proteoform-level FDR.

Mass spectrometry data was deposited to the ProteomeXchange Consortium via the PRIDE [73] partner repository with the dataset identifier PXD059108.

**HeLa Cell Culture**

Group 2. HeLa cells were cultured in MEM-containing 10% fetal bovine serum (v/v) and maintained in a humidified atmosphere of 95% air and 5% CO2 at 37 °C. The adherent cell layer was washed with PBS and then trypsinized with 0.05% trypsin-EDTA solution for 5 min at 37 °C. Then, the cells were centrifuged at 250 × g for 5 min to remove trypsin, followed by washing with PBS three times.

Group 3. HeLa-S3 cells (ATCC, Manassas, VA) were cultured in F-12K medium supplemented with 10% FBS, 100 I.U./mL penicillin, and 100 µg/mL streptomycin and converted to grow in suspension mode following a previously published protocol^[74]^ Cell count and viability were determined using a two-chip disposable hemocytometer (Bulldog Bio, Portsmouth, NH) and trypan blue staining, respectively.

***4. Statistics***

The reproducibility of CE separation across different instrumentation, conditions, and sample preparations was assessed by comparing the relative standard deviation (RSD) of migration times in the analysis of the Pierce Intact Protein Mix. Due to the drastic differences in CZE-MS-based TDP setups between these labs, particularly microchip-based vs. standard CE systems, the migration time windows ranged from 2-6 up to 35-60 mins. Using RSD values removes any setup and condition-specific differences that would be reflected in the arithmetic mean and standard deviation of the migration times for each protein; these potential differences and justification for using the RSD are highlighted in Table 5 of the Pilot Data. Additionally, the impact of internal standard correction was evaluated by normalizing the migration time of each protein observed to the migration time of either carbonic anhydrase II or thioredoxin (one of the six proteins in the sample).

**Table S1**. Constituents of Pierce^TM^ Standard Protein Mix.

| Protein | Species | UniProt ID | Sequence Length | Average Mol. Mass | Modifications | pI Value  (prim. seq.) |
| --- | --- | --- | --- | --- | --- | --- |
| Insulin-like growth factor-1 LR3 | *Homo sapiens (Human)* | P05019  (40-118) | 83 | 9,111.5 Da | 3x disulfide bonds | 8.62 |
| Insulin-like growth factor-1 LR3 (N🡪D) | *Homo sapiens (Human)* | P05019  (40-118) | 83 | 9,112.5 Da | 3x disulfide bonds  1x deamidation | 8.27 |
| Thioredoxin | *Homo sapiens (Human)* | Q99757  (60-166) | 107 | 11,865.6 Da | 1x disulfide bond | 4.88 |
| Protein G | *Streptococcus dysgalactiae* | P06654 (223-413) | 198 | 21,442.7 Da | - | 4.61 |
| Carbonic Anhydrase II | *Bos taurus (Bovine)* | P00921 | 259 | 28,981.5 Da | - | 6.58 |
| Protein AG | *Staphylococcus aureus* | P02976  P19909 | 454 | 50,460.4 Da | - | 4.65 |
| Exo Klenow Fragment | *Escherichia Coli (strain K12)* | P00582 (324-928) | 605 | 68,001.7 Da | - | 5.67 |

**Table S2**. Summary of CE instrument and experimental conditions used by study participants.

| Group | Interface Type | Interface Model | L_cap_ | ID_cap_  OD_cap_ | V_cap_ | Coating | V_Inj_ | Voltage | Supp. Press. | BGE |
| --- | --- | --- | --- | --- | --- | --- | --- | --- | --- | --- |
| 1 | Sheathless | Sciex OptiMS | 91 cm | 30 µm  150 µm | 643 nL | LPA^1^ | 6.7 nL | +15 kV | 1 psi | 3% HAc^5^ |
| 2 | Nano Sheath-flow | EMASS-II | 100 cm | 50 µm  360 µm | 1,964 nL | LPA^1^ | 50 nL | +30 kV | - | 5% HAc^5^ |
| 3 | Sheathless | Sciex OptiMS | 91 cm | 30 µm  150 µm | 643 nL | LPA^1^ | 3.3 nL | +15 kV | 1 psi | 3% HAc^5^ |
| 4 | Sheathless | Sciex OptiMS | 91 cm | 30 µm  150 µm | 643 nL | LPA^1^ | 7.5 nL | +30 kV | 2 psi | 10% HAc^5^ |
| 5 | Sheath-flow | Agilent Triple-Tube | 65 cm | 50 µm  360 µm | 1,276 nL | LPA^1^ | 5.3 nL | +25 kV | - | 50 mM HAc^5^ |
| 6 | Nano Sheath-flow | nanoCEasy | 60 cm | 50 µm  360 µm | 1,178 nL | PEO^2^ | 11.8 nL | +15 kV | - | 2 M HAc^5^ |
| 7 | Nano Sheath-flow | EMASS-II | 120 cm | 50 µm  360 µm | 2,356 nL | PVA^3^ | 172 nL | +30 kV | - | 0.1% FAc^6^ |
| 8 | Nano Sheath-flow | EMASS-II | 100 cm | 50 µm  360 µm | 1,964 nL | LPA^1^ | 50 nL | +30 kV | - | 5% HAc^5^ |
| 9 | Sheath-flow | Agilent Triple-Tube | 125 cm | 50 µm  360 µm | 2,454 nL | PVA^3^ | 50 nL | +30 kV | -5 mbar | 2.5% HAc^5^ |
| 10 | Microchip | ZipChip | 22 cm | 70 µm w.  10 µm h. | 152 nL | PEG^4^ | 1 nL | +11 kV | - | 1% FAc^6^ 50% ACN^7^ |
| 11 | Microchip | ZipChip | 22 cm | 70 µm w.  10 µm h. | 152 nL | PEG^4^ | 1 nL | + 11 kV | - | 1% FAc^6^ 50% ACN^7^ |
| 12 | Sheathless | Custom | 70 cm | 50 µm  360 µm | 1374 nL | LPA^1^ |  | - 15 kV | - | 5% HAc^5^ |

^1^LPA = linear polyacrylamide, ^2^PEO = poly(ethylene oxide), ^3^PVA = poly(vinyl alcohol), ^4^PEG = poly(ethylene glycol)

^5^HAc = acetic acid, ^6^FAc = formic acid, ^7^ACN = acetonitrile

**Table S3**. Summary of MS instruments and key experimental conditions used by study participants.

| Group | MS Type | MS Model | MS1 Resolution | Activation Type | Data-Dependent Acquisition (DDA) | MS2 Resolution |
| --- | --- | --- | --- | --- | --- | --- |
| 1 | Orbitrap | Eclipse - Tribrid | 120k / 7.5k | HCD^1^ | TopN (2 s) | 60k |
| 2 | Orbitrap | Q Exactive HF | 120k | HCD^1^ | Top5 | 60k |
| 3 | Orbitrap | Q Exactive UHMR | 6.25k | NA | NA | NA |
| 4 | QTOF | Bruker Impact | NA | NA | NA | NA |
| 5 | QTOF | Bruker maXis II | 60k | CID^2^ | Top5 | 60k |
| 6 | Orbitrap | Fusion Lumos - Tribrid | 60k | HCD^1^ | NA | 120k |
| 7 | TOF | Agilent TOF 6230 | NA | NA | NA | NA |
| 8 | Orbitrap | Q Exactive HFX | 140k | (i) HCD^1^  (ii) UVPD^3^ | Top5 | 60k |
| 9 | IM-QTOF | Agilent 6560 IM Q-TOF | NA | CID^2^ | Top5 | NA |
| 10 | Orbitrap | Exploris 240 BioPharma | 15k | HCD^1^ | (i) Top3  (ii) targeted | (i) 15k  (ii) 45k |
| 11 | TOF | Bruker timsTOF Pro | NA | CID^2^ | NA | NA |
| 12 | Orbitrap | Exploris 240 | 15k | HCD^1^ | Top3 | 60k |

^1^HCD = higher-energy collisional dissociation, ^2^CID = collision-induced dissociation, ^3^UVPD = ultraviolet photodissociation

**Table S4**. Migration order of standard proteins in various CE-MS approaches used by study participants.

| Group | Peak 1 | Peak 2 | Peak 3 | Peak 4 | Peak 5 | Peak 6 | Peak 7 |
| --- | --- | --- | --- | --- | --- | --- | --- |
| 1 | Carbonic Anhydrase II | Exo Klenow Fragment | IGF-LR3^1^ | IGF-LR3^1^ (N🡪D) | Protein AG | Protein G | Thioredoxin |
| 2 | Carbonic Anhydrase II | - | IGF-LR3^1^ | IGF-LR3^1^ (N🡪D) | Protein AG | Protein G | Thioredoxin |
| 3 | Carbonic Anhydrase II | - | IGF-LR3^1^ | IGF-LR3^1^ (N🡪D) | Protein AG | Protein G | Thioredoxin |
| 4 | IGF-LR3^1^ | Exo Klenow Fragment | Carbonic Anhydrase II | IGF-LR3^1^ (N🡪D) | Protein AG | Protein G | Thioredoxin |
| 5 | Carbonic Anhydrase II | Exo Klenow Fragment | IGF-LR3^1^ | IGF-LR3^1^ (N🡪D) | Thioredoxin | Protein AG | Protein G |
| 6 | IGF-LR3^1^ | - | Carbonic Anhydrase II | - | - | Protein G | Thioredoxin |
| 7 | Carbonic Anhydrase II | Exo Klenow Fragment | IGF-LR3^1^ | IGF-LR3^1^ (N🡪D) | Protein G | Thioredoxin | Protein AG |
| 8 | - | - | IGF-LR3^1^ | IGF-LR3^1^ (N🡪D) | - | Protein G | Thioredoxin |
| 9 | Carbonic Anhydrase II | - | IGF-LR3^1^ | IGF-LR3^1^ (N🡪D) | Protein AG | Protein G | Thioredoxin |
| 10 | IGF-LR3^1^ | IGF-LR3^1^ (N🡪D) | Exo Klenow Fragment | Carbonic Anhydrase II | Thioredoxin | Protein G | Protein AG |
| 11 | IGF-LR3^1^ | - | Exo Klenow Fragment | Carbonic Anhydrase II | Thioredoxin | Protein G | Protein AG |
| 12^2^ | Thioredoxin | Protein G | Protein AG | IGF-LR3^1^ (N🡪D) | IGF-LR3^1^ | Exo Klenow Fragment | Carbonic Anhydrase II |

^1^IGF-LR3 = Insulin-like growth factor-1 LR3, ^2^group 12 utilized reversed polarity (-kV) for separation

**Table S5**: RSD values (%) observed for standard proteins migration time via CZE-MS across triplicate measurements.

| Group | Carbonic Anhydrase II | Exo Klenow Fragment | IGF-LR3^1^ | IGF-LR3^1^ (N🡪D) | Protein AG | Protein G | Thioredoxin | Mean |
| --- | --- | --- | --- | --- | --- | --- | --- | --- |
| 1 | 2.18 | 2.17 | 2.22 | 2.27 | 2.35 | 2.43 | 2.50 | 2.30 |
| 2 | 1.68 | NA | 1.66 | 1.78 | 1.89 | 1.93 | 1.84 | 1.80 |
| 3 | 0.87 | NA | 0.78 | 0.74 | 0.77 | 0.81 | 0.88 | 0.81 |
| 4 | 0.24 | 0.19 | 0.17 | 0.17 | 0.18 | 0.18 | 0.13 | 0.18 |
| 5 | 0.76 | 1.49 | 1.86 | 1.04 | 0.93 | 0.98 | 1.11 | 1.17 |
| 6 | 0.49 | NA | 0.47 | NA | NA | 0.50 | 0.52 | 0.49 |
| 7 | 4.98 | 5.26 | 6.53 | 7.11 | 7.82 | 8.72 | 7.81 | 6.89 |
| 8 | NA | NA | 1.18 | 1.90 | NA | 1.40 | 1.90 | 1.60 |
| 9 | 2.50 | NA | 2.85 | 3.16 | 3.30 | 2.30 | NA | 2.82 |
| 10 | 1.27 | 1.07 | 1.19 | 1.18 | 1.39 | 1.38 | 1.21 | 1.24 |
| 11 | 0.37 | 0.39 | 0.34 | N/A | 0.28 | 0.23 | 0.27 | 0.31 |
| 12 | 2.53 | 0.64 | 0.55 | 0.44 | 1.21 | 0.63 | 0.89 | 0.98 |

^1^IGF-LR3 = Insulin-like growth factor-1 LR3

**Table S6**: Relative standard deviations (RSDs) (%) for standard proteins after internal standard correction using Trx for migration time normalization*.

| Group | Carbonic Anhydrase II | Exo Klenow Fragment | IGF-LR3^1^ | IGF-LR3^1^ (N🡪D) | Protein AG | Protein G | Thioredoxin | Mean |
| --- | --- | --- | --- | --- | --- | --- | --- | --- |
| 1 | 0.35 | 0.36 | 0.32 | 0.26 | 0.19 | 0.12 | - | 0.27 |
| 2 | 0.32 | NA | 0.43 | 0.27 | 0.64 | 0.22 | - | 0.38 |
| 3 | 0.04 | NA | 0.13 | 0.25 | 0.15 | 0.09 | - | 0.13 |
| 4 | 0.15 | 0.18 | 0.08 | 0.08 | 0.07 | 0.07 | - | 0.11 |
| 5 | 0.28 | 0.35 | 0.08 | 0.33 | 0.14 | 0.12 | - | 0.22 |
| 6 | 0.12 | NA | 0.07 | NA | NA | 0.04 | - | 0.08 |
| 7 | 2.94 | 2.66 | 1.33 | 0.74 | 0.18 | 0.96 | - | 1.47 |
| 10 | 0.06 | 0.15 | 0.19 | 0.19 | 0.34 | 0.17 | - | 0.18 |
| 11 | 0.15 | 0.18 | 0.13 | N/A | 0.13 | 0.08 | - | 0.13 |
| 12 | 1.38 | 0.47 | 0.31 | 0.54 | 0.72 | 0.66 | - | 0.68 |

^1^IGF-LR3 = Insulin-like growth factor-1 LR3, *Groups 8 and 9 are omitted due to a small number of proteins detected or lack of observing thioredoxin (Trx)

**Table S7.** Summary of CE instrument and experimental conditions used for the yeast protein extract.

| Group | Interface Type | Interface Model | L_cap_ | ID_cap_  OD_cap_ | V_cap_ | Coating | V_Inj_ | Voltage | Supp. Press. | BGE | Sample Buffer |
| --- | --- | --- | --- | --- | --- | --- | --- | --- | --- | --- | --- |
| 1 | Sheathless | Sciex OptiMS | 91 cm | 30 µm  150 µm | 643 nL | LPA^1^ | 60 nL | +15 kV | 0.5 psi | 3% HAc^6^ | 100 mM AmAc^9^ |
| 2 | Nano Sheath-flow | EMASS-II | 100 cm | 50 µm  360 µm | 1,964 nL | LPA^1^ | 200 nL | +30 kV | - | 5% HAc^6^ | 100 mM AmAc^9^ |
| 3 | Sheathless | Sciex OptiMS | 91 cm | 30 µm  150 µm | 643 nL | BFS^2^ | 10 nL | 15 kV | 1 psi |  | Water |
| 5 | Sheath-flow | Agilent Triple Tube | 120 cm | 50 µm 360 µm | 2,355 nL | BFS^2^ | 5 nL | +30 kV | - | 1M FAc^7^ | Water |
| 6 | Nano Sheath-flow | nanoCEasy | 150 cm | 50 µm 360 µm | 2,945 nL | PEO^3^ | 31 nL | + 30 kV | - | 10% IPA in 1M FAc^7^ | 10% IPA in 1M FAc |
| 7 | Nano-Sheath flow | EMASS-II | 120 cm | 50 µm 360 µm | 2,355 nL | PVA | 172 nL | +30 kV | - | 0.1% FAc^7^ | 25 mM AmAc^9^ |
| 11 | Microchip | ZipChip | 22 cm | 70 µm w.  10 µm d | 152 nL | PEG^4^ | 1 nL | 500 V/cm for 0-10 minutes, gradient from 500 to 650 V/cm over 10 to 15 minutes. | - | 1% FAc^7^ 50% ACN^8^ | Water |
| 12 | Sheathless | Custom | 70 cm | 50 µm 360 µm | 1374 nL | PEI^5^ | 36 nL | -15 kV | - | 5% HAc^6^ | 25 mM AmBc^10^ |

^1^LPA = linear polyacrylamide, ^2^BFS = bare-fused silica, ^3^PEO = polyethylene oxide, ^4^PEG = polyethylene glycol, ^5^PEI = polyethylene imine, ^6^HAc = acetic acid, ^7^FAc = formic acid, ^8^ACN = acetonitrile, ^9^AmAc = ammonium acetate, ^10^AmBc = ammonium bicarbonate

**Table S8.** Summary of MS instruments and key experimental conditions used for the yeast protein extract

| *Group* | *MS Type* | *MS Model* | *MS1 Resolution* | *Activation Type* | *Data-Dependent Acquisition (DDA)* | *MS2 Resolution* |
| --- | --- | --- | --- | --- | --- | --- |
| 1 | Orbitrap | Eclipse – Tribrid | 120k / 7.5k | HCD^1^ | TopN (2 s) | 60k |
| 2 | Orbitrap | Q Exactive HF | 120k | HCD^1^ | Top5 | 60k |
| 3 | Orbitrap | Orbitrap Fusion Lumos | 120k | HCD^1^ | Top5 | 60k |
| 5 | TOF | Bruker MaXis II | 60k | CID^2^ | Top5 | 60k |
| 6 | Orbitrap | Orbitrap Fusion Lumos | 120k | ETD^3^ | TopN (6s) | 60k |
| 7 | TOF | Agilent TOF 6230 | N/A | CID^2^ | Top5 | N/A |
| 11 | TOF | timsTOF pro | N/A | N/A | 5-10 Hz scan rate auto MS/MS | N/A |
| 12 | Orbitrap | Ascend Biopharma | 120k | HCD^1^ | Top5 | 60k |
| 13 | TOF | Bruker timsTOF Flex | N/A | N/A | 2 Hz scan rate auto MS/MS | N/A |

^1^HCD = High-energy C-trap dissociation, ^2^CID = collision-induced dissociation, ^3^ETD = electron-transfer dissociation

**Table S9**. The number of protein and proteoform identifications from the CZE-MS/MS analysis of the yeast lysate sample by five research groups.*

| Group | Separation | Interface Type | MS Model | Inj. Amount (ng) | Avg. Protein IDs | Avg. Proteoform IDs |
| --- | --- | --- | --- | --- | --- | --- |
| 1 | CE (Nano Sheath-flow) | Sciex OptiMS | Orbitrap Eclipse | 100 | 276±38 | 1710±168 |
| 2 | CE (Sheathless) | EMASS-II | Q Exactive HF | 160 | 213±7 | 879±26 |
| 3 | CE (Sheathless) | Sciex OptiMS | Orbitrap Fusion Lumos | 60 | 174±20 | 832±48 |
| 3 | LC | Dionex Ultimate 3000 | Orbitrap Fusion Lumos | 100 | 213±1 | 1039±11 |
| 6 | CE (Nano Sheath-flow) | NanoCEasy | Orbitrap Fusion Lumos | 31 | 56±14 | 134±35 |
| 12 | CE (Sheathless) | Custom | Orbitrap Ascend | 18 | 122±2 | 474±25 |

*The average protein and proteoform numbers from the triplicate measurement are listed. The LC-MS/MS data from group 3 are also listed.

**Table S10.** Summary of CE and MS conditions for HeLa cell lysate analysis

| Group | *CE type* | *BGE* | *Inj. Amount* | *MS Model* | *MS1 Resolution* | *Activation Type* | *Data-Dependent Acquisition (DDA)* | *MS2 Resolution* |
| --- | --- | --- | --- | --- | --- | --- | --- | --- |
| 2 | Sheath flow | 5% Acetic Acid | 100 ng | Orbitrap Exploris 480 | 480k | HCD | Top6 | 60k |
| 3 | Sheathless | 20% Acetic Acid | 20 ng | Orbitrap Fusion Lumos | 120k | HCD | Top5 | 60k |


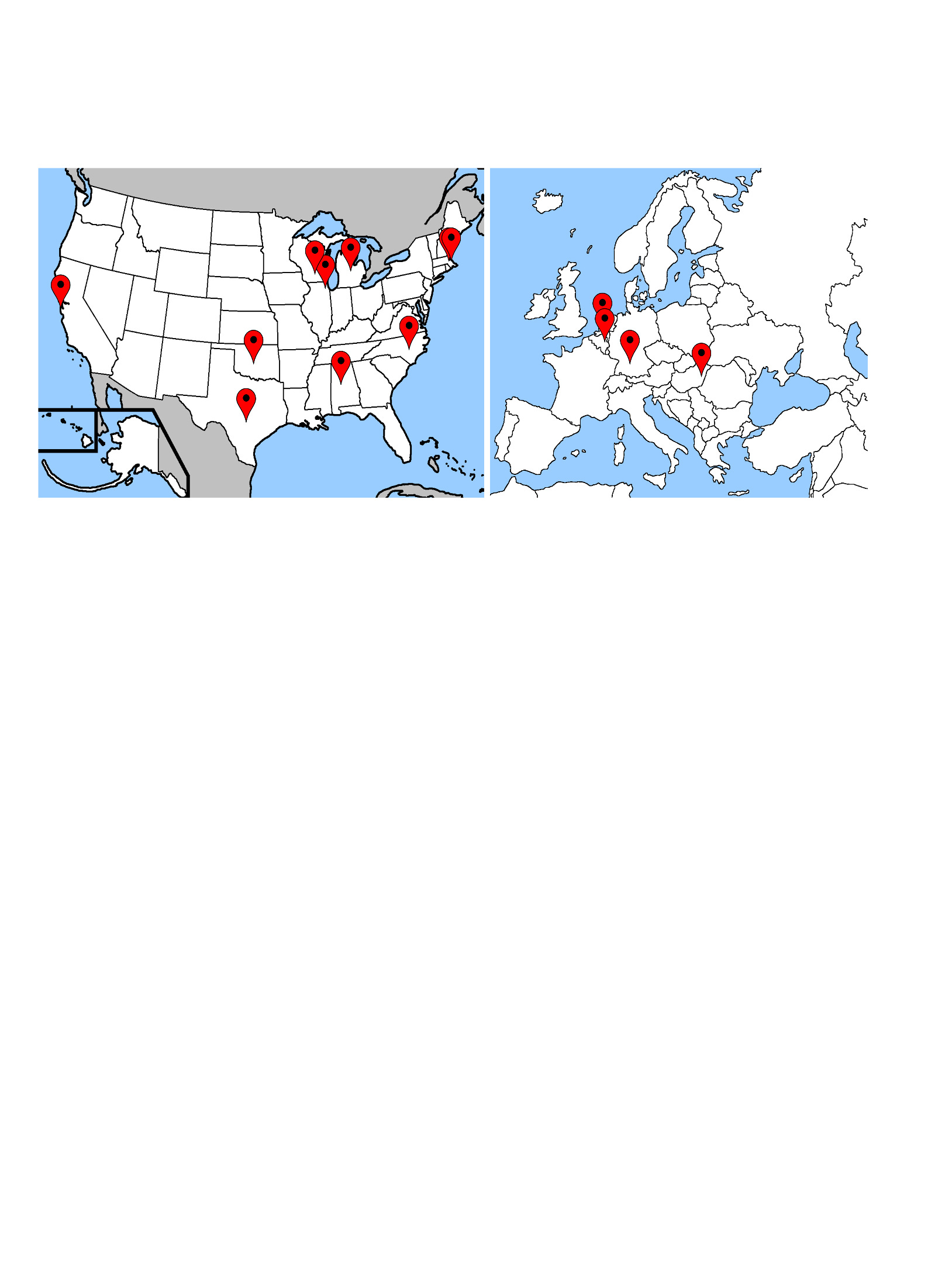


**Figure S1.** Map of the location of study participants, with each pin representing the location of the group(s). One research group has two locations on the map, which is why the figure has 13 labels.


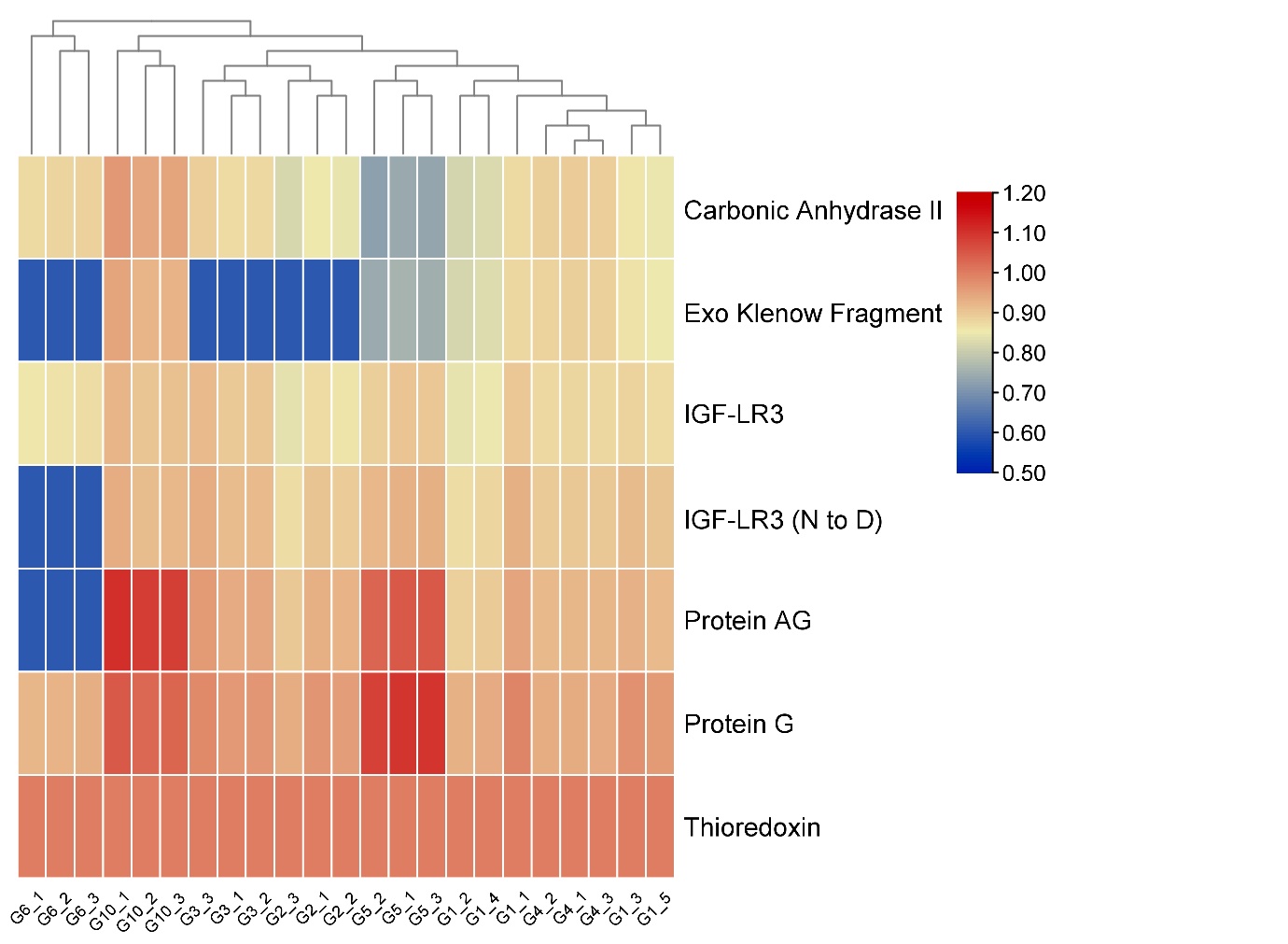


**Figure S2.** Unsupervised hierarchical clustering analysis conducted using the observed migration times for the Pierce^TM^ intact protein mix from different groups (G1_1 = Group 1, run 1). Migration times were normalized to the migration time of thioredoxin, and missing values (unidentified proteins) were given an imputed value of 0.5.


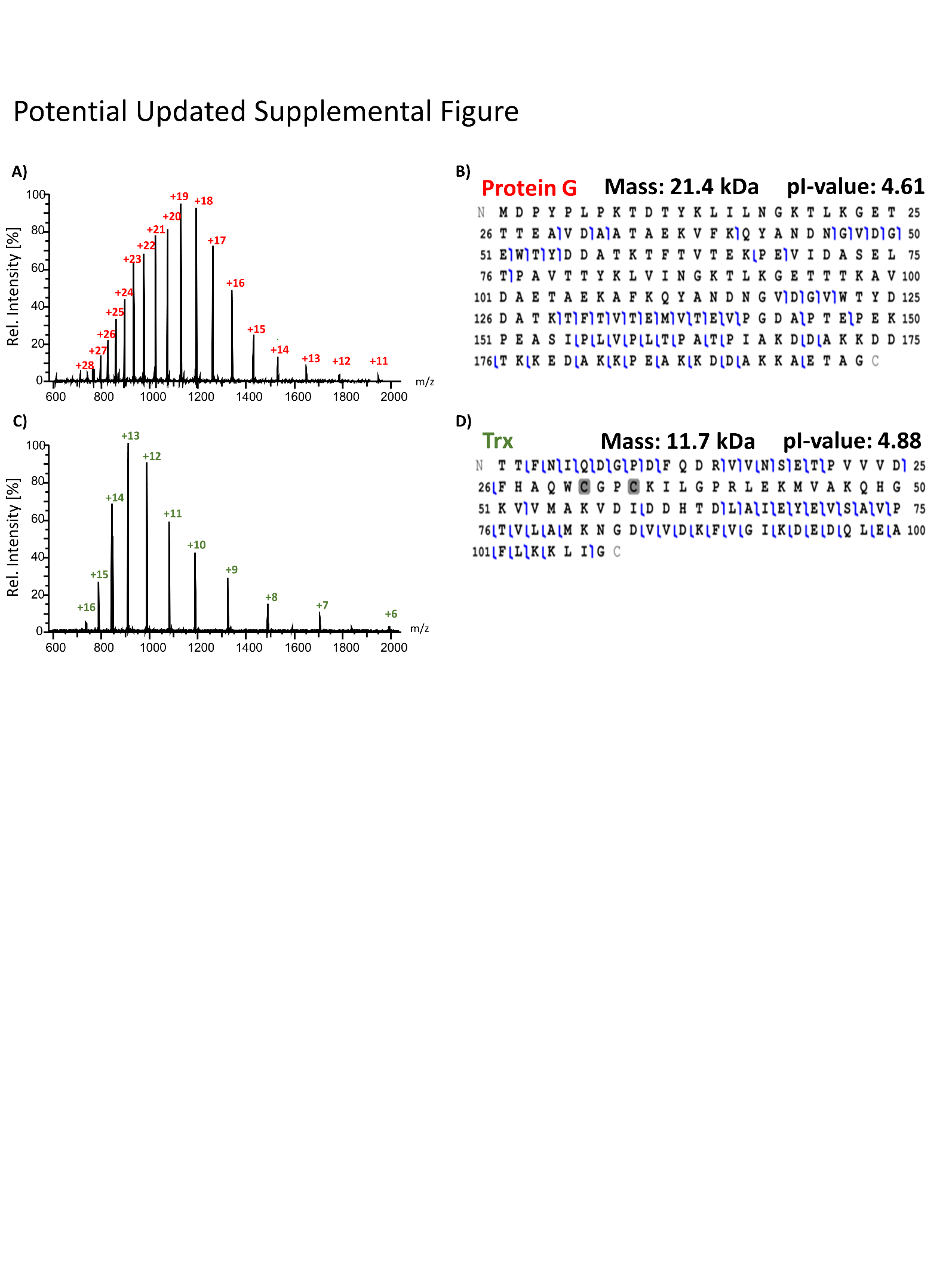


**Figure S3.** Representative mass spectra and fragmentation maps of protein G and thioredoxin (A) mass spectrum of protein G, (B) fragmentation map for MS/MS analysis of protein G, (C) mass spectrum of thioredoxin (D) fragmentation map for MS/MS analysis of thioredoxin. The fragmentation map for thioredoxin (D) exhibits low coverage in the center region due to the intact disulfide bond (residues highlighted in grey) stabilizing this region.


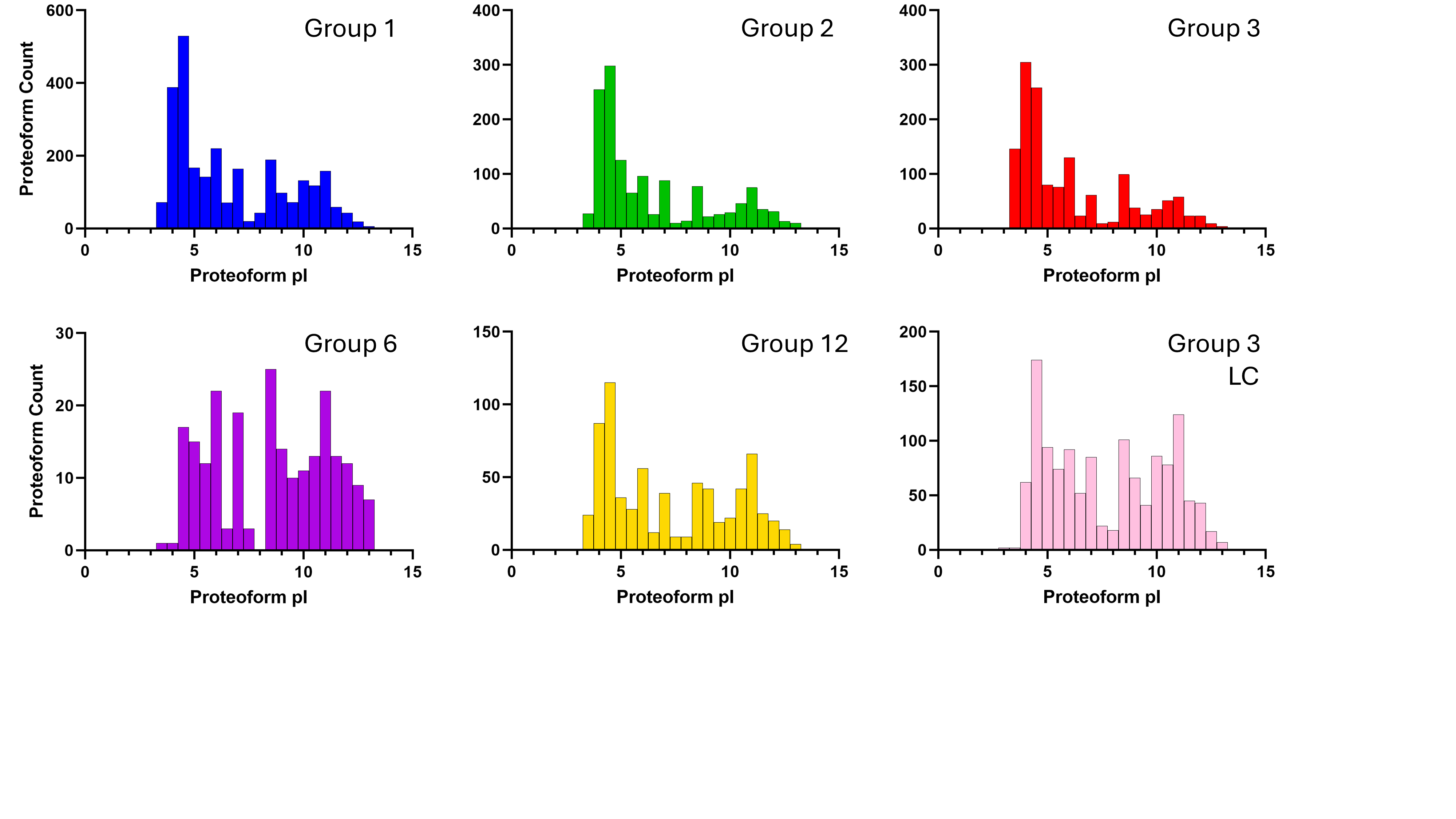


Figure S4. pI distributions of observed yeast proteoforms from five Groups of CE participants and LC data provided by Group 3.


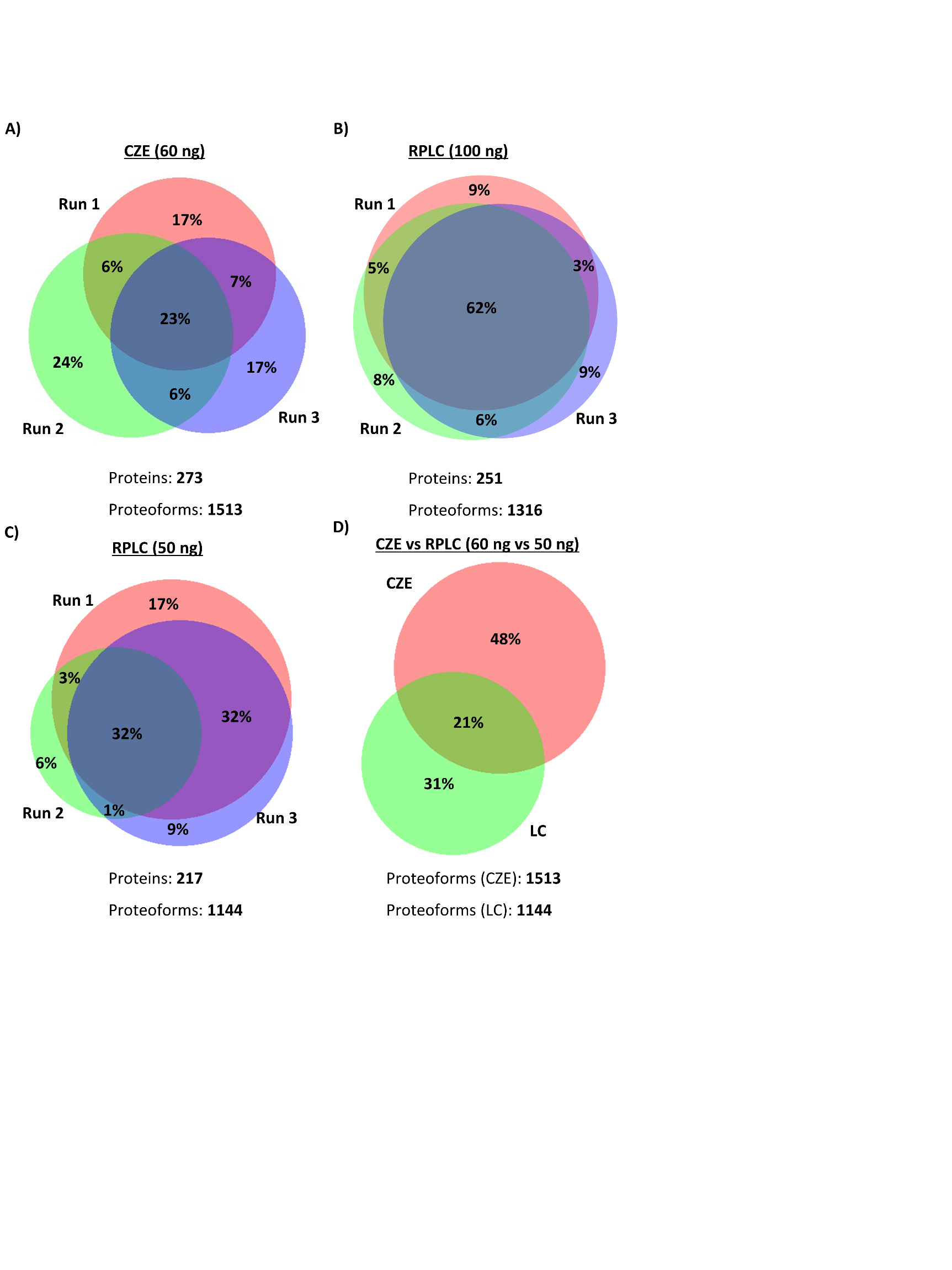


**Figure S5.** Comparison of CZE and LC protein and proteoform analysis of yeast lysate. (A) CZE analysis of 60 ng of yeast lysate (Group 3) with total identified proteins and proteforms. (B) LC analysis of 100 ng of yeast lysate (Group 3) with total identified proteins and proteoforms. (C) LC analysis of 50 ng of yeast lysate (Group 3) with total identified proteins and proteoforms. Run 2 observed a marked decrease in identified proteoforms, which, based on the observed decrease in the total ion chromatogram for the run, was likely due to an injection issue. (D) Overlap in identified proteoforms between CZE at 60 ng and LC at 50 ng, which shows that 48% of proteoforms observed across the CZE and LC runs (both N=3) were uniquely observed only via CZE analysis. All runs were performed on the same mass spectrometer (Orbitrap Fusion Lumos) with the same MS and MS/MS parameters, using the same bulk prepared sample.


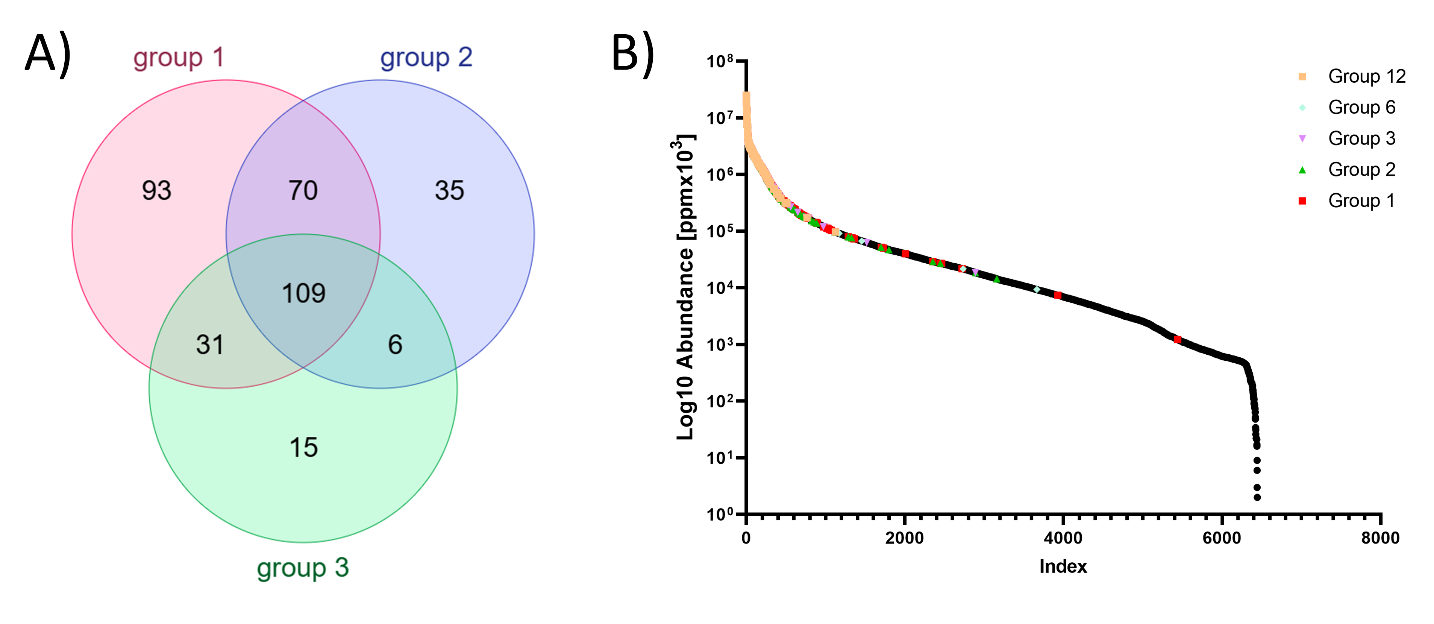
**Figure S6.** Comparison of observed yeast proteins from the top-down proteomic analysis of the Promega intact yeast lysate standard. A) Venn diagram of the overlap in observed proteins for the yeast lysate standard from groups 1, 2, and 3. These groups were chosen due to their similar sample loading amounts. B) S-curve of protein abundance per cell from the PaxDb integrated protein abundance database for S. Cerevisiae^[75]^, with the observed yeast proteins from each group marked on the curve.


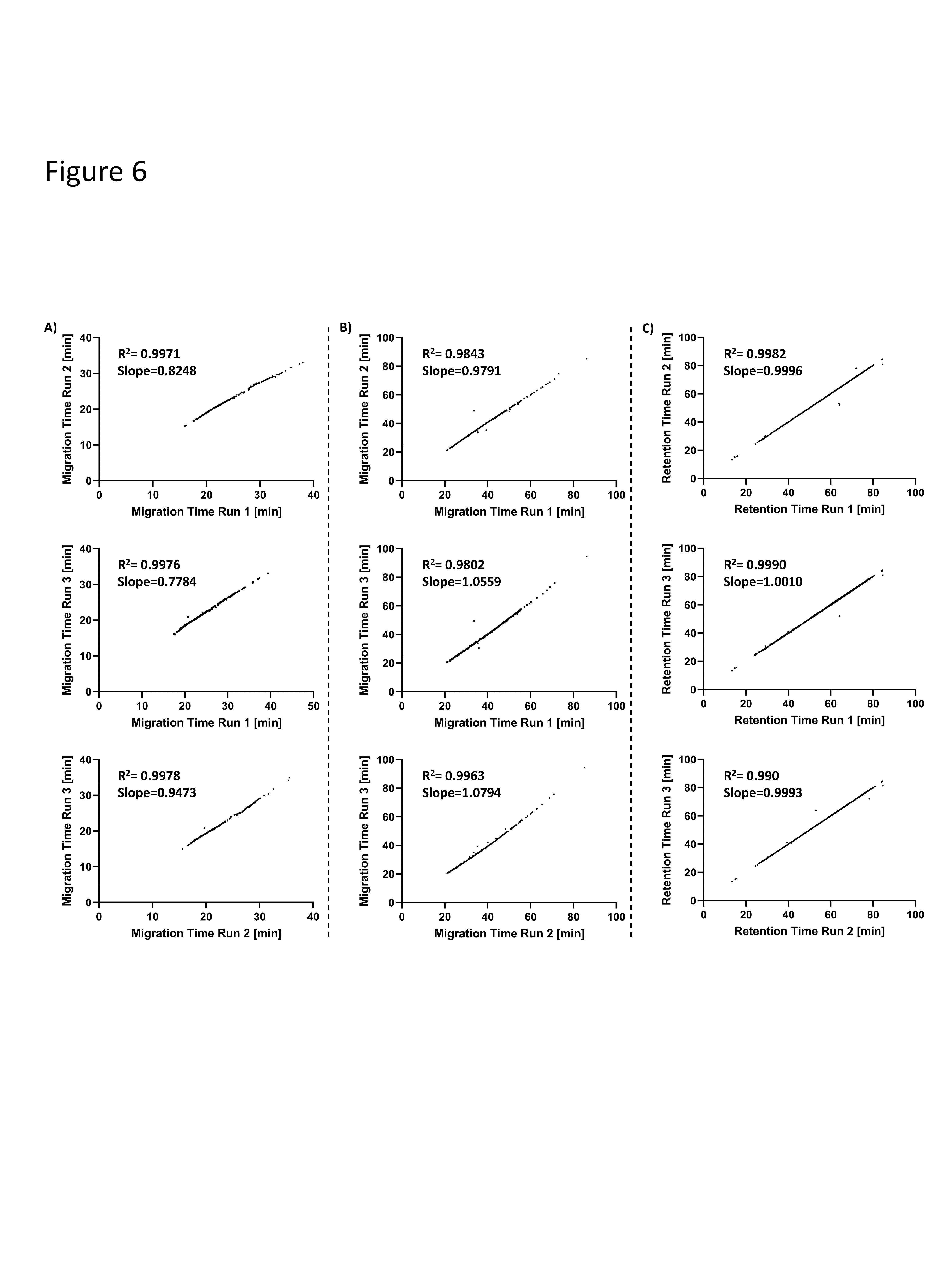


**Figure S7**. Migration time and retention time run-to-run reproducibility for observed proteoforms in analyses of the yeast lysate sample. A) Migration time reproducibility for observed proteoforms from Group 12, where a positively coated PEI capillary was used with 18 ng of sample loaded. B) Migration time reproducibility for observed proteoforms from Group 2, where a neutrally coated LPA capillary was used with 160 ng of sample loaded. C) Retention time reproducibility for observed proteoforms from Group 3, where an in-house packed C4 column was used with 100 ng of sample loaded.


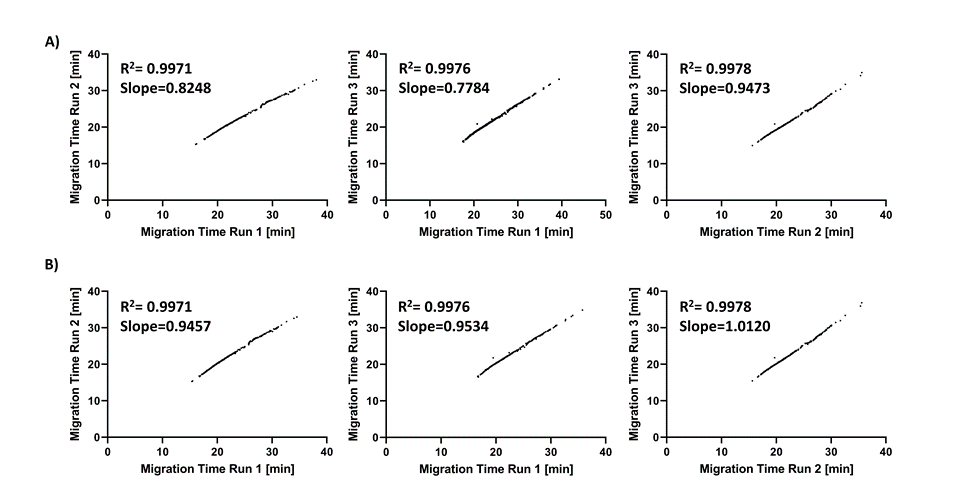


**Figure S8**. Comparison of CE-MS migration time reproducibility for CE TDP-MS analysis of the yeast cell lysate sample from Group 12 before (A) and after (B) migration time correction. B) Migration times in the runs shown in (A) were adjusted using a two-point internal correction via proteoforms observed in all three runs at the beginning and end of the migration window for detected proteoforms.

***References***

[78] S. H. Abdeen, A. M. Abdeen, H. A. El-Enshasy, A. A. El Shereef, “HeLa-S3 Cell Growth Conditions in Serum-Free Medium and Adaptability for Proliferation in Suspension Culture”, *Journal of Biological Sciences* **2011**, *11(2),* 124-1314

[79] Q. Huang, D. Szklarczyk, M. Wang, M. Simonovic, C. von Mering, “PaxDb 5.0: Curated Protein Quantification Data Suggests Adaptive Proteome Changes in Yeasts”, *Mol Cell Proteomics* **2023**, 22(10)
